# Supplementary material for: Intervention targeted at physicians’ treatment of musculoskeletal disorders and sickness certification: an interrupted time series analysis
Source: BMJ Open. 2021 Dec 2;11(12):e047018. doi: 10.1136/bmjopen-2020-047018 (PMC8647396; doi:10.1136/bmjopen-2020-047018)
Supplement: Supplementary data [file bmjopen-2020-047018supp001.pdf]

Low back (lumbar back) pain, lumbago, M54.5

Lumbago is a very common ailment that everyone experiences at some point in their lives. It involves pain, aching and stiffness in the lumbar back. **It is almost always a harmless and benign condition**, which gets better by itself, but often recurs in following months. However, even if it recurs, its prognosis is good. The pain may begin suddenly in a harmless situation but may also develop gradually.

- **As a rule, lumbago gets better in a few days**. For more than 90% of sufferers, back pain is completely gone within 4–6 weeks.
- Signs of serious back disorders (continuous and progressively increasing backache even at rest, fever, weight loss, high-energy trauma, osteoporosis, changes in urinary and bowel continence, cancer history) can be ruled out using simple methods.

Assessing the need for sick leave:

o According to new recommendations, sick leave may not at all necessary. There is no medical evidence that physically strenuous work slows down the healing of ordinary back pain or that it is associated with a risk of the condition continuing or of further injury.

o **If sick leave is deemed necessary**: The patient's ability to do physically light work (only some lifting, bending and turning movements) may be **reduced for a few days or a week at the most.**

o The patient's ability to perform physically strenuous work (which includes lifting, bending and turning movements) may be reduced for **two weeks at the most** (+ 2 weeks of partial sick leave if necessary), mainly because of difficulty lifting heavy objects. **Only after this does the checklist below apply.**

- **Temporary reduction and modification of pain-provoking factors are recommended.**
- Despite low back pain, working is not dangerous, and as a rule, continuing to work and not avoiding physical strain speeds up healing.
    - **Patients should be advised to use their backs normally – this is not dangerous**. Pain may become prolonged if the patient remains passive and afraid to move. Physical activity is beneficial; excessive resting is harmful. Moderate pain during exercise is not dangerous.
    - It is advisable to initially treat pain with sufficiently high doses of paracetamol and/or anti-inflammatory drugs. Strong painkillers (opiates) or muscle relaxants have not shown to be beneficial.
    - If the pain becomes prolonged, manipulation, massage or acupuncture may be beneficial.
    - In cases of prolonged pain, diverse drug-free pain management and pain threshold medicine can reduce pain sensitivity and improve pain tolerance.
    - Sleep and mood problems related to pain should be treated.

Kausto J, *et al. BMJ Open* 2021; 11:e047018. doi: 10.1136/bmjopen-2020-047018

Checklist for when perceived work disability becomes prolonged:

DIAGNOSIS:

- Have **inflammatory diseases** (e.g., ankylosing spondylitis: Marie-Strümpell disease) been ruled out?
- Could the reason for the pain be a significant back **injury or previous cancer**?
- Does the pain or numbness radiate below the knee (**sciatic nerve entrapment)**?

WORK:

- Has work been modified to **temporarily reduce repeated lifting, leaning forward and twisted back postures, and work tasks that cause vibration**?
- Have p**sychosocial work load factors** (poor workplace atmosphere, lack of support from supervisor, excessive time pressure, overly demanding work, etc.) been identified? If so, has any attempt been made to change them?
- Has the employee **spoken to their supervisor** about their work and work ability?
- Have **occupational health services** been in contact with the supervisor **about work arrangements**?
- Has an **occupational physiotherapist** visited the workplace to advise on how to modify work tasks?

INDIVIDUAL FACTORS:

- Does the patient have any loading factors **outside of work** that may affect pain tolerance and coping with pain (crisis, grief, prolonged stress)?
- Does the patient do any **activities or** have any **hobbies outside of work** that may maintain the pain (sports, home renovations, long-distance driving, frequent use of laptop or mobile phone, gardening)?

TREATMENT:

- Have the **treatments** that the patient feels have best **benefit** been determined? Which treatments **make the pain worse**?
- Has the patient received **self-care instructions**?
- Does the patient use **active means** to control their pain? Exercise?

These issues, ‘yellow flags’, increase the risk of prolonged pain and work disability in the case of back pain:

- Sleep problems
- Anxiety, depression
- Fear of permanent damage or worsening of pain
- Pain all over the body
- Passivity, avoidance behaviour
- Problems at work, poor job satisfaction
