# Supplementary material for: Intervention targeted at physicians’ treatment of musculoskeletal disorders and sickness certification: an interrupted time series analysis
Source: BMJ Open. 2021 Dec 2;11(12):e047018. doi: 10.1136/bmjopen-2020-047018 (PMC8647396; doi:10.1136/bmjopen-2020-047018)
Supplement: Author's manuscript [file bmjopen-2020-047018.draft_revisions.pdf]

BMJ Open

BMJ Open is committed to open peer review. As part of this commitment we make the peer review history of every article we publish publicly available.

When an article is published we post the peer reviewers' comments and the authors' responses online. We also post the versions of the paper that were used during peer review. These are the versions that the peer review comments apply to.

The versions of the paper that follow are the versions that were submitted during the peer review process. They are not the versions of record or the final published versions. They should not be cited or distributed as the published version of this manuscript.

BMJ Open is an open access journal and the full, final, typeset and author-corrected version of record of the manuscript is available on our site with no access controls, subscription charges or pay-per-view fees (http://bmjopen.bmj.com).

If you have any questions on BMJ Open's open peer review process please email info.bmjopen@bmj.com

BMJ Open

The effectiveness of an intervention targeted at physicians' treatment of musculoskeletal disorders and sickness certification: An interrupted time series analysis

Journal:	*BMJ Open*
Manuscript ID	bmjopen-2020-047018
Article Type:	Original research
Date Submitted by the Author:	17-Nov-2020

Complete List of Authors:	Kausto, Johanna; Finnish Institute of Occupational Health<br>Rosenström, Tom Henrik; University of Helsinki Faculty of Medicine, Department of Psychology and Logopedics<br>Ervasti, Jenni; Finnish Institute of Occupational Health<br>Pietiläinen, Olli; University of Helsinki, Department of Public Health<br>Kaila-Kangas, Leena; Finnish Institute of Occupational Health<br>Rahkonen, Ossi; University of Helsinki, Department of Public Health<br>Harkko, Jaakko; University of Helsinki Faculty of Social Sciences<br>Vaananen, Ari; Finnish Institute of Occupational Health<br>Kouvonen, Anne; University of Helsinki, Faculty of Social Sciences; Queen's University Belfast, Administrative Data Research Centre<br>Lallukka, Tea; University of Helsinki, Department of Public Health

Keywords:	OCCUPATIONAL & INDUSTRIAL MEDICINE, PUBLIC HEALTH, Musculoskeletal disorders < ORTHOPAEDIC & TRAUMA SURGERY

SCHOLARONE™
Manuscripts

I, the Submitting Author has the right to grant and does grant on behalf of all authors of the Work (as defined in the below author licence), an exclusive licence and/or a non-exclusive licence for contributions from authors who are: i) UK Crown employees; ii) where BMJ has agreed a CC-BY licence shall apply, and/or iii) in accordance with the terms applicable for US Federal Government officers or employees acting as part of their official duties; on a worldwide, perpetual, irrevocable, royalty-free basis to BMJ Publishing Group Ltd ("BMJ") its licensees and where the relevant Journal is co-owned by BMJ to the co-owners of the Journal, to publish the Work in this journal and any other BMJ products and to exploit all rights, as set out in our licence.

The Submitting Author accepts and understands that any supply made under these terms is made by BMJ to the Submitting Author unless you are acting as an employee on behalf of your employer or a postgraduate student of an affiliated institution which is paying any applicable article publishing charge ("APC") for Open Access articles. Where the Submitting Author wishes to make the Work available on an Open Access basis (and intends to pay the relevant APC), the terms of reuse of such Open Access shall be governed by a Creative Commons licence – details of these licences and which Creative Commons licence will apply to this Work are set out in our licence referred to above.

Other than as permitted in any relevant BMJ Author's Self Archiving Policies, I confirm this Work has not been accepted for publication elsewhere, is not being considered for publication elsewhere and does not duplicate material already published. I confirm all authors consent to publication of this Work and authorise the granting of this licence.

For peer review only

BMJ OPEN
Public health research

The effectiveness of an intervention targeted at physicians' treatment of musculoskeletal disorders and sickness certification: An interrupted time series analysis

Johanna Kausto[1], Tom Rosenström[1, 2], Jenni Ervasti[1], Olli Pietiläinen[3], Leena Kaila-Kangas[1], Ossi Rahkonen[3], Jaakko Harkko[4], Ari Väänänen[1], Anne Kouvonen[4,5], Tea Lallukka[3]

1 Finnish Institute of Occupational Health, Helsinki, Finland

2 Department of Psychology and Logopedics, Faculty of Medicine, University of Helsinki, Helsinki, Finland

3 Department of Public Health, University of Helsinki, Helsinki, Finland

4 Faculty of Social Sciences, University of Helsinki, Helsinki, Finland

5 Administrative Data Research Centre - Northern Ireland, Queen's University Belfast, Belfast, UK

*Corresponding author:

Johanna Kausto

Specialized Researcher

Finnish Institute of Occupational Health
PO Box 18
00032 TYÖTERVEYSLAITOS

FINLAND

johanna.kausto@ttl.fi

tel. + 358 30 474 2509, + 358 43 8200 698

Counts: Abstract (272), main text (4257), Tables (3), Figures (2)

Keywords: Sick leave [MeSH], Practice guidelines [MeSH], Occupational Health Service [MeSH], Musculoskeletal Diseases [MeSH]

For peer review only

Abstract

Objectives: We investigated the effectiveness of the intervention on employee sick leave applying interrupted time-series analysis.

Design: Register-based cohort study with a quasi-experimental study design.

Setting: Employees of the City of Helsinki, Finland (approximately 38,000 annually, with annual turnover of about 10%).

Participants: We analysed individual-level register-based data on all employees who were employed by the City for any length of time between 2013 and 2018 (a total 86,970 different employees and 3,014,075 sick leave days). Sick leave days and periods that were occupational health services (OHS) -based constituted the intervention time series and the rest of the sick leave days and periods contributed to the control time series.

Intervention: An intervention providing recommendations for physicians on managing pain and prescribing sick leave for low back, shoulder, and elbow pain was carried out at the OHS of the City of Helsinki beginning in 2016.

Outcome measures: Number of sick leave days per month and sick leave periods per year.

Results: For all sick leave days prescribed in OHS, a gradual intervention effect decreasing the number of sick leave days was detected. On average, the intervention was estimated to have saved 2.5 sick leave days per year per employee, amounting to approximately 25 million EUR per year as direct cost. For other sick leave days, there was both an immediate increase in the level of sick leave days after the intervention and a subsequent gradual effect of decreasing the number of sick leave days.

Conclusions: The intervention reduced employee sick leaves and therefore it is likely that it had led to direct cost savings. However, further evidence for causal inferences is needed.

For peer review only - http://bmjopen.bmj.com/site/about/guidelines.xhtml

Article Summary

Strengths and limitations of this study

- This study presents novel information on the effectiveness of introducing recommendations for management of pain and prescribing sick leave for musculoskeletal disorders on employee sick leave.

- Comprehensive individual level data from employer registers were linked to individual level data from national registers.

- We applied interrupted time series analysis with a control time series which is a strong quasi-experimental design, recommended for examining population-level interventions.

-Intervention status was not obtainable for the entire follow-up time, and information on diagnoses was available only for sick leave periods that lasted over 11 calendar days.

For peer review only

For peer review only - http://bmjopen.bmj.com/site/about/guidelines.xhtml

Introduction

Musculoskeletal disorders (MSD) are highly prevalent among working age population, and they result in extensive disability costs both for the individuals and the society. In Finland in 2019, 27% of the days of sick leave periods lasting at least 11 calendar days were due to MSD [1]. Thus, sick leave is typically granted for musculoskeletal pain symptoms, such as back, shoulder, or elbow pain. However, scientific evidence does not support the beneficiality of long sick leaves. For example, it has been shown that when having low back pain, from a point of view of recovery, staying active is better than bed rest [2, 3].

Primary care doctors' advice can have a major impact on whether an individual takes sick leave and for how long [4]. In a follow-up study it was found that visiting a medical specialist because of a musculoskeletal disorder was associated with a delayed full return to work irrespective of the severity of ailment [5]. Furthermore, earlier studies have found that sick listing practices vary a lot across physicians [6, 7]. A Finnish study showed that the differences in the length of prescribed sick leaves cab be up to 8-fold [8]. In line with this, a survey found that Finnish physicians experienced that they needed more information, guidance and training on sick listing [9].

Against this background, an intervention was carried out at Occupational Health Helsinki, which provides occupational health services (OHS) for the City of Helsinki employees. The intervention was launched in 2016. Introducing and increasing the use of shared guidelines on the management of pain and work disability and prescribing sick leave for low back,

For peer review only

For peer review only - http://bmjopen.bmj.com/site/about/guidelines.xhtml

shoulder and elbow pain was the central element in the intervention. To investigate the effectiveness of this intervention on employee sick leaves, we carried out prospective controlled time series and binomial regression analyses using register-based data.

Methods

Study design and population

Data were collected as a part of the Helsinki Health Study [10]. The study population includes a sample of the employees of the City of Helsinki. Women represent 70% of the employees who work in general local administration, social and health care, education and culture, public transport, and technical services. All employees share the same personnel administration, policies, and occupational health services [10]. Employees can use the services (including primary care services for other than work-related conditions) of their OHS provider, Occupational Health Helsinki; or they can seek treatment from public or private health care. The employees of the City of Helsinki can use self-certification for sick leaves lasting up to five days. In the case of self-certification, a medical certificate is not needed, but the employee notifies the supervisor when taken ill. In 2016, self-certification was most common among young employees (under 30 years of age) [11].

For the purposes of this study, individual-level register-based data of all the employees who were employed by the City for any length of time between 2013 and 2018 (n= 86,970) were analysed. Background information on participants were drawn from employer’s registers and information on all sick leave periods (start and end dates and diagnostic codes (ICD-10)) during this time were derived from employer’s registers and the national sickness insurance

For peer review only - http://bmjopen.bmj.com/site/about/guidelines.xhtml

register of the Social Insurance Institution of Finland (SII). ICD-10 codes for sick leave periods were available from the SII for sick leave periods lasting more than 11 calendar days. Data on chronic somatic and psychiatric illnesses and the purchases of prescribed psychotropic medication were obtained from the Register of Special Reimbursement for Medication Purchases (SII). Data from these three registers were linked based on unique individual numbers of the participants. Such numbers are assigned to each citizen of Finland at birth and for migrants when they get a residence permit, and they are common to all administrative registers, enabling extensive record linkages [12]. The study follows the Helsinki Health Study (HHS) protocol in line with the University of Helsinki's guidelines and the EU and Finnish data legislation. The ethics committees of the Department of Public Health, the University of Helsinki and the health authorities of the City of Helsinki have approved the HHS study. The City of Helsinki and register holders have given permissions for data linkage.

Patient and public involvement

Patients or the public were not involved in the design, or conduct, or reporting, or dissemination plans of our research.

Study context

Occupational Health Care in Finland

In the Finnish occupational health service (OHS) system all employees are entitled to statutory preventive OHS provided by the employer. Most employers offer state-subsidised free primary care services for their employees on top of the statutory preventive OHS. OHS

For peer review only - http://bmjopen.bmj.com/site/about/guidelines.xhtml

providers in Finland provide primary care services to employees in a way that is comparable to primary care in international comparisons in substance, but differs in that the service is targeted only to the working population [13]. The statutory tasks of OHS include assessment of the health and safety aspects of the work, assessment and monitoring of employees' health and work ability, making initiatives for improvement and monitoring their implementation, advice and guidance, monitoring employees with disabilities and referring them to rehabilitation, co-operation with representatives of other health care services and social insurance, participation in organising first aid at the workplace, participation in activities that maintain work ability, and monitoring the quality and impact of occupational health care activities [14].

Intervention

Occupational Health Helsinki provides occupational health care to the almost 40,000 employees working annually in the City departments. The employees represent hundreds of different occupations, and a rather large supply of health care services supporting the employees' work ability and the functioning of work units is offered. The Occupational Health Helsinki staff consists of 150 health care professionals.

The aims of the intervention were to increase the quality and effectiveness of occupational health care, to increase the use of mutually created and shared guidelines based on available scientific evidence in assessing work disability, to provide a practical instrument for physicians in managing pain and prescribing sick leave in the form of recommendations, to help physicians to take into account the complexity of the association between pain and work disability, and to provide up-to-date information to the patient about their pain

For peer review only - http://bmjopen.bmj.com/site/about/guidelines.xhtml

condition, its treatment and how it may affect work and work ability. The goal was to reduce employee sickness absence and improve work ability.

Recommendations on work disability management and prescribing sick leave for low back pain, shoulder pain and elbow pain were launched first in January-2016 and they were the central and the most noticeable component in the intervention. Recommendations were launched in a form of three guideline papers (one for each diagnostic category). Training regarding the implementation of the guidelines was organised in OHS for physicians and other relevant health care professionals such as physiotherapists and nurses, and an intensified follow-up of the sick leave trends was started in OHS. For instance, recent trends of sick leave were reported in the monthly meetings for the physicians.

As an example, in the case of low back pain, the recommendation emphasised that the condition is very common and nearly all people experience a disabling low back pain (i.e. pain, ache and stiffness in the area of lower back) at some point in their lives. The condition is usually benign in nature, heals spontaneously in a few days, and 90% of the patients will fully recover in 4 to 6 weeks. Still, possible serious causes for back pain should be ruled out. In sick listing the following points should be considered: In many cases sick leave is not needed; There is no indication that physically strenuous work would slow down recovery; If sick listing is necessary, a few days up to a week should be sufficient in light duties; If work includes heavy lifting, bending or rotation, two weeks of full time sick leave may be needed; Factors that provoke pain should be temporarily modified; Factors that suggest an increased risk for prolonged back pain and work disability include sleeping problems, depression and anxiety, fear avoidance beliefs, multisite pain, and problems at the workplace and

For peer review only

For peer review only - http://bmjopen.bmj.com/site/about/guidelines.xhtml

dissatisfaction with work. The recommendations included a checklist for the physicians to be used at the appointment.

Intervention status

Sick leave periods that were OHS-based (if a sick leave was preceded by a likely related appointment at OHS within 11 days, it was approximated that sick-listing had taken place at OHS) constituted the intervention time series and the rest of the sick leave periods (no appointment at OHS, but appointment and sick-listing had taken place elsewhere or the employee had used self-certification, in which case there had been no medical appointment at all) contributed to the control time series. The available registers contained information of appointments in the OHS until 20th April 2017.

Outcome

We calculated the number of sick leave days per month and sick leave periods per year.

Covariates

Covariates that were available in the registers and were either important descriptive factors of the sick leave time series or were regarded as potential confounders included age, sex, occupational class (based on occupational title and categorised as upper grade non-manual employees, intermediate level non-manual employees, lower grade non-manual employees and manual workers), job contract (permanent, temporary) valid 1st January 2016, chronic somatic illnesses (derived from the Register of Special Reimbursement for Medication Purchases, and included diabetes, heart disease, rheumatoid arthritis, chronic asthma, stage 2 hypertension, Parkinson’s disease, epilepsy, uremia, bowel disease, multiple sclerosis

For peer review only - http://bmjopen.bmj.com/site/about/guidelines.xhtml

disease, and diseases of pancreas and categorized as 0=no, 1=yes) valid 1st January 2016, and purchases of prescribed psychotropic medication (Anatomic Therapeutic Chemical, ATC, classification N06 or N05 categorised as 0=no, 1=yes) between 1st November 2015 and 1st November 2016.

Statistical analyses

We used an interrupted time series (ITS) design with a control time series [15-17]. The observational unit in the analyses was a sick leave day or period. The ITS analysis uses time-series (a continuous sequence of observations on a population-level taken repeatedly at equal intervals over time) for a specific outcome to establish an underlying trend which is "interrupted" by an intervention at a specific point of time. A strength of ITS analyses is that they are generally not affected by typical confounding factors (such as age distribution or socioeconomic status) as they model a time trend in a context where the population composition (in terms of the confounding factors) remains rather constant before vs. after the intervention. Measured time varying confounders can be controlled for in the regression models. Unmeasured or unknown time varying confounders can be controlled by adding a control group (not exposed to the intervention) [15]. It has been recommended that the covariate balance between the intervention and control time series is explored, though it is not a prerequisite for the analysis [16].

Our integer-valued (count, or proportion) outcome variable was days of sick leave per month (range 0–30, or from 0 to days exposed within the 30-day period, if not with the employer for the full 30-day period), recorded separately for each month. Although each employee was followed up for multiple months, different employees may have different

For peer review only - http://bmjopen.bmj.com/site/about/guidelines.xhtml

time average rate of sick leave days, introducing statistical dependencies to the observations. As our interests pertained to a population-level causal effect, within employee clustering in the outcome was considered a nuisance variable and its effects were modeled using generalised estimating equations [18]. Given the data characteristics above, the outcome was modeled using generalised (binomial) linear regression model and the models were estimated with generalised estimating equations. Although our data was in the form of monthly counts, an exponentiated binomial regression coefficient can be interpreted as an odds ratio (OR) for a sick-leave day (vs. no sick leave) for a randomly chosen day, adjusting for the other covariates in the model. As a sensitivity analysis, we also tested quasi-binomial generalised linear model with usual maximum likelihood estimation, which gave similar results with narrower confidence intervals.

The main test of our hypothesis pertained to the model equation

$$g^{-1}(Y) = \beta_0 + \beta_{slope}(m - m_0) + \beta_{immediate}X + \beta_{gradual}X(m - m_0),$$

where Y is the outcome, g the logit-link function, X is 1 for all months after the intervention launch date (1st of January 2016) and 0 before it, and $(m - m_0)$ is time (months, centered around the time of intervention [19] , m_0). Here, β_{slope} is the linear trend effect before the intervention, whereas $\beta_{slope} + \beta_{gradual}$ is the trend after the intervention took place. The intercept before the intervention is β_0 and that after the intervention $\beta_0 + \beta_{immediate}$. Thus, $\beta_{immediate}$ is the immediate intervention effect and $\beta_{gradual}$ the gradual intervention effect that accumulates at the rate $\beta_{gradual}$ per month after the intervention (for the remaining observation period). Besides this usual ITS setting, we took into account the annual fluctuation of sick leave by modelling an annual trend estimating coefficients for two

For peer review only - http://bmjopen.bmj.com/site/about/guidelines.xhtml

additional covariates with values $\sin(m2\pi/12)$ and $\cos(m2\pi/12)$, where the factor $2\pi/12$ scales monthly data points m to the annual cycles.

Because our outcome data timepoints (months) could include sick leave days from prescribed at OHS and other sources, defining a covariate for intervention group membership would have required us to prioritise either OHS or other sources. Instead of formal testing of such covariate, we conducted separate analyses for the intervention and control groups. Both crude models and models adjusted for the covariates were fitted. Analyses were run separately for men and women (who are well known to differ in sick leave behaviour), for short (<= 11 calendar days) and longer (> 11 calendar days) sick leave periods, and for sick leave periods (>=11 calendar days) in the diagnostic categories of back pain (ICD-10 M54.5), shoulder pain (ICD-10 M75) and elbow pain (ICD-10 M77.1).

As the sick leave recommendations potentially had an effect both on the number of sick leave days and periods, the number of consecutive sick leave periods were computed within index month plus 5 immediately preceding months. Monthly sample averages of numbers of periods were investigated with otherwise similar linear models as in above but using a six-month moving-average error structure. The “arima” function of R version 3.5.1 (2018-07-02) was used. The same software was used for all the analyses.

Results

Descriptive results

Our data included information on sick leaves of a total of 86,970 employees covering more than 73 months. Due to workforce turnover, the monthly sample size of employees

For peer review only - http://bmjopen.bmj.com/site/about/guidelines.xhtml

averaged at 41,289, with a minor variance across months (min 37,567, max 45,520; SD 2,054). Overall, 87% of all employees were on sick leave at least once during the follow-up and 33% of the employees were prescribed sick leave at OHS at least once between 1st January 2013 and 21st April 2017. Of those employees who were prescribed sick leave at OHS, 95% were prescribed sick leave also elsewhere (or they used self-certification). Of those who were prescribed sick leave elsewhere (or used self-certification) at least once, 46% were prescribed sick leave also at OHS. Of all employees, 30% were not on sick leave at all. On average, employees were prescribed 0.36 days of sick leave per month at OHS and 0.82 sick leave days per month were prescribed elsewhere or were self-certified. The respective mean numbers of sick leave periods per employee per year were 0.49 and 1.96.

Descriptive statistics for all employees, employees with sick leaves prescribed at OHS and employees with other sick leaves (prescribed elsewhere or self-certificated) are shown in Table 1 and Table 2. Those employees who were prescribed sick leave at OHS at least once by 21st April 2017, were more often permanently employed, older, female, and intermediate or lower level non-manual workers (Table 1).

Monthly averages of the number of all sick leave days

Since it is generally recommended to draw simple plots of ITS analyses before entering into more complex model fitting [15, 16], we started by examining the monthly average days of sick leave per employee. Based on a visual inspection of monthly averages, the number of all sick leave days prescribed at OHS declined shortly after the intervention had started in 2016 (Figure 1a). This might reflect a causal effect of the intervention because similar temporal changes were not observed for other sick leave periods (Figure 1b). After 21st April

For peer review only - http://bmjopen.bmj.com/site/about/guidelines.xhtml

2017, our register data could not differentiate between sick leave days that were OHS-based and other sick leave days, but we had a longer stretch of data when counting in all sick leaves. A possible intervention effect was visible in a visual inspection of all sick leaves (thick line for local-regression smoother), as well as annual periodic variation (thin dashed line in Figure 1c). When removing cyclic annual variation and a linear trend, the visual suggestion of an intervention effect remained. However, sick leaves appeared to return to preceding rates towards the end of the longest available follow-up data (in 2018) (Figure 1d).

Formal modeling (adjusting for seasonal variation) did not reveal an immediate intervention effect ($\beta_{immediate}$ = 0.01, 95% CI = (-0.052–0.072)), but did detect a gradual intervention effect ($\beta_{gradual}$ = -0.007, 95% CI = (-0.013– -0.001)) on the number of OHS-based sick leave days. The corresponding numbers were similar in direction but not statistically significant in the control group ($\beta_{immediate}$ = 0.068, 95% CI = (-0.065–0.201)), and ($\beta_{gradual}$ = -0.009, 95% CI = (-0.022– 0.004)) (data not shown). These formal findings would support a gradual causal effect of intervention, but we were able to achieve a greater statistical power by accessing the employee-level data instead of the above-discussed monthly averages.

Results on employee-level data: The number of all sick leave days

We modelled the number of sick leave days using binomial regression on monthly sick leave days per employee. Figures 2 a and b illustrate the model predictions for the number of all sick leave days. For convenient illustration, we plotted the model estimates overlaid on the above discussed monthly averages, but importantly these models use 2,160,445 observations from 75,962 individuals instead of 52 monthly averages. The exact binomial

For peer review only - http://bmjopen.bmj.com/site/about/guidelines.xhtml

regression coefficients (with logit-link function) as well as odds ratios for a sick leave day at index month vs. preceding month are presented in Table 3.

We observed a clear gradual diminishing intervention effect in the number of all-cause OHS-based sick leave days (first model, Table 3). A similar gradual trend was found in the control group (sick-listing elsewhere or self-certification), but that was offset by an increase in the level of the number of sick leave days at the time of intervention. Adjusting for the covariates (Table 3, model 2) did not affect the estimates significantly. We additionally ran the analyses separately for men and women (not shown in the table). The results were in line with the first model (total sample). The intervention effect found in all-cause sick leave was not paralleled in diagnostic group-specific findings (models 4 to 6 in Table 3).

While a gradual intervention effect of odds ratio 0.986 may appear small, we can illustrate the effect of the intervention in a practical way as follows. Assuming employees on average have 1.2 sick-leave days per month (cf. Fig. 1c), their baseline risk rate per day would be 0.04. Small risk ratios closely match odds ratios. Thus, if the other factors have already been adjusted for, the intercept for a logistic model would be $\beta_0 = \log(0.04)$. Then the gradual intervention effect of $OR_{gradual} = 0.986$ would imply that odds ratio of a sick-leave day one year after the intervention has reduced to $\exp(\beta_0 + 12 \times \log(OR_{gradual})) \approx 0.034$ and the risk rate of a sick-leave day to approximately 0.033. Thus, the gradual intervention effect on average amounts to $(0.04 - 0.033) \times 30 \times 12 = 2.52$ avoided sick-leave days per year per employee. Expressed in another way, the intervention may have saved 2,520 sick-leave days per year per 1,000 employees. A company with 40,000 employees and a direct cost of approximately 250 EUR per sick-leave day (numbers similar to those of the City of Helsinki),

For peer review only - http://bmjopen.bmj.com/site/about/guidelines.xhtml

would therefore save $2520 \times 40 \times 250$ EUR, amounting to approximately 25 million EUR per year.

Results on the employee-level data: The number of all sick leave periods

As a sensitivity analysis, we investigated the number of all-cause sick leave periods as an outcome. A sick leave period is a consecutive spell of sick leave days. We report results in units of periods per year per employee, although we computed periods per index month plus five preceding months (Figure 2 c and d). We analysed the monthly averages with a moving-average regression model. For all sick leave periods prescribed in OHS, we did not detect any immediate effect of intervention ($\beta_{immediate}$ = 0.017, 95% CI = (-0.011– 0.046)), but a gradual intervention effect diminishing the number of sick leave periods was found ($\beta_{gradual}$ = -0.009, 95% CI = (-0.013– -0.005)). The findings on the gradual effect were similar in the control group ($\beta_{gradual}$ = -0.014, 95% CI = (-0.026– -0.003)), whereas the immediate effect indicated that an increase of the sick leave periods occurred in the control group at the time of intervention ($\beta_{immediate}$ = 0.141, 95% CI = (0.067– 0.214)).

Discussion

In this register-based study among municipal employees, we analysed sick leave trends before and after an intervention targeted at physicians' management of musculoskeletal pain-related work disability and prescribing sick leave. The intervention was carried out in OHS setting. We examined monthly averages of all-cause sick leave days and found no immediate intervention effect among OHS-based sick leave days but did detect a gradual

For peer review only - http://bmjopen.bmj.com/site/about/guidelines.xhtml

intervention effect decreasing the number of OHS-based sick leave days by 2.5 days per year per employee. The corresponding findings were similar in direction but not statistically significant for the control time series (sick-listing elsewhere or self-certification). In the employee-level data (with a larger statistical power), we found a clear gradual intervention effect decreasing the number of OHS-based all-cause sick leave days. Similar gradual decreasing trend was found for the control time series, but that was offset by an increase in the level of sick leave days and periods at the time of intervention.

The knowledge base on physicians' sick-listing practices is accumulating. The present study suggests that the intervention affected the sick-listing practices of physicians working in Occupational Health Helsinki. There was a downward trend in the number of OHS-based sick leave days and periods. Nevertheless, the findings may also suggest that soon after the intervention carried out in OHS, the employees increasingly sought treatment elsewhere (or used self-certification increasingly) as a rise in the level of other sick leaves was detected. This trend leveled down, however.

There are a great number of previous studies on interventions attempting to change physicians' behaviour in different areas of clinical practice. Findings show that success in changing the behaviour and adherence to different clinical guidelines varies. Some studies report success in behaviour change [20, 21] whereas other studies report limited success or found interventions ineffective [22-24]. Moreover, a change in physician's behaviour does not automatically reflect in patient outcomes [23]. Guidelines have been found to be a necessary but an insufficient step in changing clinical care [25]. The importance of proactive

For peer review only - http://bmjopen.bmj.com/site/about/guidelines.xhtml

implementation of guidelines and efforts to include the new practice into existing organisational procedures, which both were attempted in this intervention, have been emphasised to achieve sustainable results [26, 27]. As the context of the intervention is important, the results of an intervention in one environment cannot directly be applied to another context.

A strength of this study is its design based on register data on all employees. We could link comprehensive data from employer registers to data from national registers using personal identification numbers. Applied ITS analysis with a control time series has been recommended for analysing population-level interventions [15, 28]. A limitation was that the intervention status was approximated (an OHS-based sick leave was preceded by a likely relevant appointment at OHS) and the information was not available for the entire follow up period. The validity of the control group can be debated [17] as all employees could be prescribed sick leave both at OHS and elsewhere (or use self-certification in short sick leaves). However only the OHS professionals were exposed to the intervention. Because the intervention was targeted at managing pain and prescribing sick leave from the first day of work disability, a limitation was that we had access to diagnostic data only for sick leave periods that lasted over 11 calendar days. This, together with the low base rate for specific diagnoses, may explain the result that the intervention effect found in among all-cause sick leaves was not paralleled in diagnostic group-specific findings of sick leaves (lasting over 11 calendar days) due to low back, shoulder, and elbow pain. Musculoskeletal disorders constitute a major cause for sick leave. The fact that an intervention effect was seen among all-cause sick leaves (regardless of the length of the sick leave period) may also indicate that

For peer review only

For peer review only - http://bmjopen.bmj.com/site/about/guidelines.xhtml

recommendations given for these three specific disorders influenced how the physicians managed work disability and prescribed sick leave altogether.

The recommendations for physicians constituted chronologically the first and the main element in the intervention. Direct access to physiotherapists without a doctor's referral was introduced in the following year, and thereafter it was not possible to differentiate the effects of these different elements.

A gradual effect of the intervention on reducing the number of sick leave days and periods prescribed in OHS was observed, but the causality of the relationship is unclear due to similar findings for the control outcome of other sick leaves. The estimated reduction of 2.5 sick leave days per year per employee could leave to savings of 25 million euros for a large employer such as the City of Helsinki. This can be considered as an economically relevant intervention effect, but further evidence for its causal interpretation is needed.

For peer review only

For peer review only - http://bmjopen.bmj.com/site/about/guidelines.xhtml

Figure legends

Figure 1: Model fits. Monthly averages of the number of all sick leave days. Intervention status could be specified until 21st April 2017 (Figures a) and b)). In Figures c) and d) the intervention status is not differentiated. Vertical dashed lines indicate the time point of intervention (all figures). Circles denote to observed averages, thick lines to their local regression fits (smoothed averages) (all figures), and thin dashed line to annual periodic variation (Figure c)).

Figure 2: Model fits. Monthly averages of the number of all sick leave days (figures a) and b)) and periods (figures c) and d)). Observed data points denote to simple monthly averages and the lines represent GEE-model fits to employee-level data. The dashed line represents the full model prediction, including annual variations, whereas the solid line is the ITS-part of the model.

ITS=Interrupted time series analysis; GEE= generalised estimation equation

Acknowledgements: We would like to thank MD, Chief Medical Officer Eevamaija Tuovinen; MD, PhD Helena Miranda; and CEO, PhD Tiina Pohjonen, Occupational Health Helsinki for providing information of the intervention and its context.

Conflicts of Interest: The authors have no conflicts of interest to declare. The funders had no role in the design of the study; in the collection, analyses, or interpretation of data; in the writing of the manuscript, or in the decision to publish the results.

Funding: This work was supported by the Social Insurance Institution of Finland (grant # 9/26/2019 JK, TR, JE, AV and LK-K), The Finnish Work Environment Fund (grant #190081 JK, TR, JE, AV and LK-K), the Academy of Finland (grants #1294514 and #319200 OR and TL) and the Economic and Social Research Council (ESRC) (grant # ES/S00744X/1 AK).

Open access funding provided by the Finnish Institute of Occupational Health.

Author Contributions: Conceptualisation and methodology: JK, TR, OP, AV, EJ, OR, JH, AK, and TL; writing–original draft preparation: JK and TR; writing–review and editing: JK, TR, EJ, OP, LKK, OR, JH, AV, AK, TL; project administration: JK.

All authors have read and agreed to the published version of the manuscript.

"We used the **STROBE cohort checklist** [29] when writing our report "The effectiveness of an intervention targeted at physicians' treatment of musculoskeletal disorders and sickness certification: An interrupted time series analysis".

Ethics approval and consent to participate: The ethics committees of the Department of Public Health, the University of Helsinki and the health authorities of the City of Helsinki approved the Helsinki Health Study protocol. The ethical approval applies to the current study. The participants gave written informed consent to participate in the study.

For peer review only - http://bmjopen.bmj.com/site/about/guidelines.xhtml

Provenance and peer review: Not commissioned; externally peer reviewed.

Data sharing statement: No additional data are available.

Licence statement: I, the Submitting Author has the right to grant and does grant on behalf of all authors of the Work (as defined in the below author licence), an exclusive licence and/or a non-exclusive licence for contributions from authors who are: i) UK Crown employees; ii) where BMJ has agreed a CC-BY licence shall apply, and/or iii) in accordance with the terms applicable for US Federal Government officers or employees acting as part of their official duties; on a worldwide, perpetual, irrevocable, royalty-free basis to BMJ Publishing Group Ltd ("BMJ") its licensees and where the relevant Journal is co-owned by BMJ to the co-owners of the Journal, to publish the Work in BMJ Open and any other BMJ products and to exploit all rights, as set out in our licence.

The Submitting Author accepts and understands that any supply made under these terms is made by BMJ to the Submitting Author unless you are acting as an employee on behalf of your employer or a postgraduate student of an affiliated institution which is paying any applicable article publishing charge ("APC") for Open Access articles. Where the Submitting Author wishes to make the Work available on an Open Access basis (and intends to pay the relevant APC), the terms of reuse of such Open Access shall be governed by a Creative Commons licence – details of these licences and which Creative Commons licence will apply to this Work are set out in our licence referred to above.

For peer review only

For peer review only - http://bmjopen.bmj.com/site/about/guidelines.xhtml

Table 1. Descriptive statistics for all employees, employees with sick leaves prescribed at OHS[1] and employees with sick leave prescribed elsewhere or self-certified (other sick leaves)*.

Characteristic	Sick-listing at OHS (n= 25200)	Other sick leaves (n=52174)	All (n=75962)
Age (Mean, SD)	44.67 (12.32)	42.16 (13.02)	40.27 (14.08)
Men (%)	20	23	27
Occupational class (%)			
Upper grade non-manual employees	21	24	27
Intermediate level non-manual employees	27	25	22
Lower grade non-manual employees	37	36	34
Manual workers	16	15	17
Job contract (%)			
Permanent	82	67	54
Prescribed reimbursed purchases of medication for chronic diseases (%)			
Yes	21	17	16
Prescribed reimbursed psychotropic medication (%)			
Yes	12	9	8

* Note that many employees were prescribed sick leave both at OHS and elsewhere (or used self-certification).

OHS=Occupational health services.

For peer review only

For peer review only - http://bmjopen.bmj.com/site/about/guidelines.xhtml

Table 2. Monthly average numbers of sick leave days per month per employee by intervention status (years 2013–2017).

	Sick-listing at OHS[1]			Other sick leaves		
	Average	Range	SD	Average	Range	SD
All sick leave days	0.380	0–30	2.59	0.840	0–30	3.40
All short sick leave periods[a]	0.117	0–25	0.79	0.392	0–24	1.24
Sick leave for low back pain[b]	0.005	0–30	0.36	0.007	0–30	0.39
Sick leave for shoulder pain[b]	0.020	0–30	0.71	0.014	0–30	0.59
Sick leave for elbow pain[b]	0.004	0–30	0.28	0.002	0–30	0.19

OHS=Occupational health services

[a] l<= 11 calendar days, [b] > 11 calendar days.

For peer review only - http://bmjopen.bmj.com/site/about/guidelines.xhtml

Table 3. Binomial regression models predicting the number of sick leave days.

	Sick listing at OHS				Other sick leaves			
	Coefficient β	Std. error	OR[1)]	95% CI	Coefficient β	Std. error	OR[1)]	95% CI
Sick leave days (all periods, all DC:s) (Crude model)[2)] (n=2 160 445 employee-months, n=75,962 employees)								
Immediate intervention effect	0.012	0.035	1.012	0.95–1.08	0.060**	0.018	1.062	1.03–1.10
Gradual intervention effect	-0.014***	0.003	0.986	0.98–0.99	-0.008***	0.002	0.992	0.99–1.00
Linear trend before intervention (Slope)	0.006***	0.001	1.006	1.00–1.01	0.002***	0.001	1.002	1.00–1.00
Sick leave days (all periods, all DC:s) (Fully adjusted model)[2)] (n=2 148 566 employee-months, n= 74,701 employees)								
Immediate intervention effect	-0.003	0.032	0.997	0.94–1.06	0.057**	0.018	1.059	1.02–1.10
Gradual intervention effect	-0.010**	0.003	0.990	0.98–1.00	-0.007***	0.002	0.993	0.99–0.99
Linear trend before intervention (Slope)	0.007***	0.001	1.007	1.01–1.01	0.003***	0.001	1.003	1.00–1.01
Sick leave days (short periods, < 11 calendar days, all dg:s) [2)] (n=2 160 445 employee-months, n=75,962 employees)								
Immediate intervention effect	0.105***	0.024	1.111	1.06–1.16	0.196***	0.010	1.217	1.19–1.24
Gradual intervention effect	-0.020***	0.002	0.980	0.98–0.98	-0.009***	0.001	0.991	0.99–0.99
Linear trend before intervention (Slope)	0.001	0.001	1.001	1.00–1.00	-0.003***	<0.001	0.997	1.00–1.00
Sick leave days in low back pain (periods >= 11 calendar days) [3)] (n=2 160 445 employee-months, n=75,962 employees)								
Immediate intervention effect	-0.547	0.340	0.579	0.30–1.13	-0.205	0.283	0.815	0.47–1.42
Gradual intervention effect	0.040	0.035	1.041	0.97–1.12	-0.019	0.031	0.981	0.92–1.04
Linear trend before intervention (Slope)	-0.004	0.009	0.996	0.98–1.01	-0.004	0.007	0.996	0.98–1.01

OHS=Occupational health services, DC=diagnostic code. OR=Odds ratio, CI=Confidence interval. *p < 0.05, **p < 0.01, *** p< 0.001.

[1)] Immediate intervention effect: Odds ratio was estimated for any employee to have any sick leave day after vs. before the intervention. Gradual intervention effect: Odds ratio was estimated for any employee to have a sick leave day at index month vs. preceding month during the period after the intervention.[2)] Follow-up from 1st January 2013 to 21st April 2017 [3)] Follow-up from 1st January 2013 to 31st December 2019. Adjusted for age, sex, occupational class, job contract, prescribed purchases of medication for chronic diseases, prescribed psychotropic medication.

For peer review only - http://bmjopen.bmj.com/site/about/guidelines.xhtml

Table 3. Binomial regression models predicting the number of sick leave days (Continued).

	Sick-listing at OHS				Other sick leaves			
	Coefficient β	Std. error	OR[1)]	95% CI	Coefficient β	Std. error	OR[1)]	95% CI
Sick leave days for shoulder pain (periods >= 11 calendar days)[3)] (n=2 160 445 employee-months, n=75,962 employees)								
Immediate intervention effect	0.365	0.207	1.441	0.96–2.16	-0.426	0.292	0.653	0.34–1.16
Gradual intervention effect	-0.023	0.023	0.977	0.93–1.02	0.013	0.025	1.013	0.97–1.06
Linear trend before intervention (Slope)	-0.004	0.008	0.996	0.98–1.01	-0.003	0.008	0.997	0.98–1.01
Sick leave days for elbow pain (periods >= 11 calendar days)[3)] (n=2 160 445 employee-months, n=75,962 employees)								
β immediate	-0.964	0.514	0.381	0.14–1.04	-0.398	0.486	0.672	0.26–1.74
β gradual	-0.034	0.072	0.967	0.84–1.11	-0.079	0.084	0.924	0.78–1.09
Slope	0.003	0.014	1.003	0.98–1.03	-0.001	0.018	0.999	0.96–1.04

OHS=Occupational health services, DC=diagnostic code. OR=Odds ratio, CI=Confidence interval. *p < 0.05, **p < 0.01, *** p< 0.001.

[1)] Immediate intervention effect: Odds ratio was estimated for any employee to have any sick leave day after vs. before the intervention. Gradual intervention effect: Odds ratio was estimated for any employee to have a sick leave day at index month vs. preceding month during the period after the intervention.[2)] Follow-up from 1st January 2013 to 21st April 2017 [3)] Follow-up from 1st January 2013 to 31st December 2019. Adjusted for age, sex, occupational class, job contract, prescribed reimbursed purchases of medication for chronic diseases, prescribed reimbursed psychotropic medication.

For peer review only - http://bmjopen.bmj.com/site/about/guidelines.xhtml

For peer review only

References

1. *Tilastotietokanta Kelasto (Statistics in Finnish)*. [cited 2020 20.10.]; Available from: https://www.kela.fi/kelasto.
2. van Tulder, M., et al., *Chapter 3. European guidelines for the management of acute nonspecific low back pain in primary care.* Eur Spine J, 2006. **15 Suppl 2**: p. S169-91.
3. Costa-Black, K.M., et al., *Back pain and work.* Best Pract Res Clin Rheumatol, 2010. **24**(2): p. 227-40.
4. Black, C.M., *Sickness absence and musculoskeletal disorders.* Rheumatology (Oxford), 2012. **51**(2): p. 204-5.
5. Lotters, F.J., M. Foets, and A. Burdorf, *Work and health, a blind spot in curative healthcare? A pilot study.* J Occup Rehabil, 2011. **21**(3): p. 304-12.
6. Englund, L., G. Tibblin, and K. Svardsudd, *Variations in sick-listing practice among male and female physicians of different specialities based on case vignettes.* Scand J Prim Health Care, 2000. **18**(1): p. 48-52.
7. Arrelov, B.E., L. Borgquist, and K.F. Svardsudd, *Influence of local structural factors on physicians' sick-listing practice: a population-based study.* Eur J Public Health, 2005. **15**(5): p. 470-4.
8. Kankaanpää, A., *Sick leave prescribing practices in Finland. Turun yliopiston julkaisuja. Annales Universitatis Turkuensis D 1132*. 2014: Turku.
9. Hinkka, K., M. Niemelä, and I. Autti-Rämö, *Sairauspoissaolotarpeen määrittäminen. Kyselytutkimus lääkäreille. (Assessing need for sick leave. A survey for physicians)*, in *Työpapereita 96*. 2016, Kela: Helsinki.
10. Lahelma, E., et al., *Cohort profile: the Helsinki Health Study.* Int J Epidemiol, 2013. **42**(3): p. 722-30.
11. Sumanen, H., et al., *Age-group differences in sickness absence among the employees of the City of Helsinki 2002–2016 (Article in Finnish).* Finnish Medical Journal 2018. **73**(7): p. 438-44.
12. Gissler, M. and J. Haukka, *Finnish health and social welfare registers in epidemiological research.* Norsk Epidemiologi, 2004. **14**(1): p. 113-120
13. Harkko, J., et al., *Socioeconomic Differences in Occupational Health Service Utilization and Sickness Absence Due to Mental Disorders: A Register-Based Retrospective Cohort Study.* Int J Environ Res Public Health, 2020. **17**(6).
14. Martimo, K.-P. and J. Mäkitalo, *The status of occupational health services in Finland and the role of the Finnish Institute of Occupational Health in the development of occupational health services. Report for the international evaluation of the Finnish Institute of Occupational Health (FIOH)*. 2014, Finnish Institute of Occupational Health: Helsinki.
15. Lopez Bernal, J., S. Cummins, and A. Gasparrini, *Interrupted time series regression for the evaluation of public health interventions: a tutorial.* Int J Epidemiol, 2017. **46**(1): p. 348-355.
16. Lopez Bernal, J., S. Cummins, and A. Gasparrini, *The use of controls in interrupted time series studies of public health interventions.* Int J Epidemiol, 2018. **47**(6): p. 2082-2093.
17. Degli Esposti, M., et al., *Can synthetic controls improve causal inference in interrupted time series evaluations of public health interventions?* Int J Epidemiol, 2020.
18. Hojsgaard, S., U. Halekoh, and J. Yan, *The R Package Geepack for Generalized Estimating Equations.* Journal of Statistical Software, 2006. **15**(2): p. 1-11.
19. Xiao, H., O. Augusto, and B.H. Wagenaar, *Reflection on modern methods: a common error in the segmented regression parameterization of interrupted time-series analyses.* Int J Epidemiol, 2020.

For peer review only - http://bmjopen.bmj.com/site/about/guidelines.xhtml

20. Bussieres, A.E., et al., *Impact of imaging guidelines on X-ray use among American provider network chiropractors: interrupted time series analysis.* Spine J, 2014. **14**(8): p. 1501-9.
21. Leece, P., et al., *Improving opioid guideline adherence: evaluation of a multifaceted, theory-informed pilot intervention for family physicians.* BMJ Open, 2020. **10**(1): p. e032167.
22. French, S.D., et al., *Evaluation of a theory-informed implementation intervention for the management of acute low back pain in general medical practice: the IMPLEMENT cluster randomised trial.* PLoS One, 2013. **8**(6): p. e65471.
23. Al Zoubi, F.M., et al., *The effectiveness of interventions designed to increase the uptake of clinical practice guidelines and best practices among musculoskeletal professionals: a systematic review.* BMC Health Serv Res, 2018. **18**(1): p. 435.
24. Zhelev, Z., et al., *Effectiveness of interventions to reduce ordering of thyroid function tests: a systematic review.* BMJ Open, 2016. **6**(6): p. e010065.
25. Timmermans, S. and A. Mauck, *The promises and pitfalls of evidence-based medicine.* Health Aff (Millwood), 2005. **24**(1): p. 18-28.
26. McCluskey, S., A.K. Burton, and C.J. Main, *The implementation of occupational health guidelines principles for reducing sickness absence due to musculoskeletal disorders.* Occup Med (Lond), 2006. **56**(4): p. 237-42.
27. Buyle, F., et al., *Implementation of guidelines for sequential therapy with fluoroquinolones in a Belgian hospital.* Pharm World Sci, 2010. **32**(3): p. 404-10.
28. Shadish, W.R., T.D. Cook, and D.T. Campbell, *Quasi-experimental experiments: Interrupted time-series designs*, in *Experimental and quasi-experimental designs for generalized causal inference*. 2002, Houghton Mifflin Company: Boston, New York. p. 623.
29. von Elm, E., et al., *The Strengthening the Reporting of Observational Studies in Epidemiology (STROBE) Statement: guidelines for reporting observational studies.* Int J Surg, 2014. **12**(12): p. 1495-9.

For peer review only

For peer review only - http://bmjopen.bmj.com/site/about/guidelines.xhtml

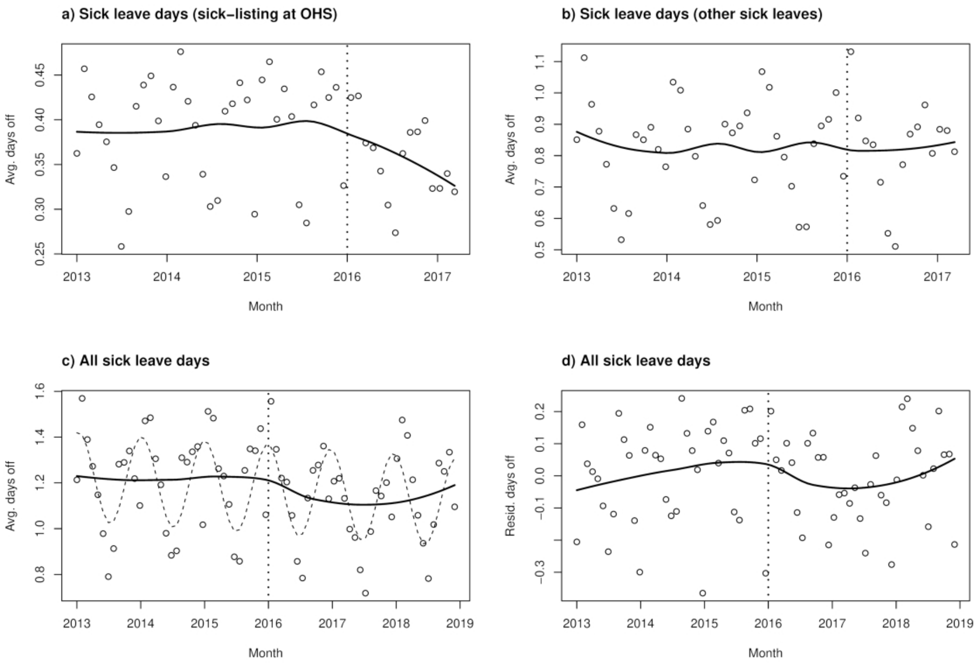

Figure 1: Model fits. Monthly averages of the number of all sick leave days. Intervention status could be specified until 21st April 2017 (Figures a) and b)). In Figures c) and d) the intervention status is not differentiated. Vertical dashed lines indicate the time point of intervention (all figures). Circles denote to observed averages, thick lines to their local regression fits (smoothed averages) (all figures), and thin dashed line to annual periodic variation (Figure c)).

173x120mm (600 x 600 DPI)

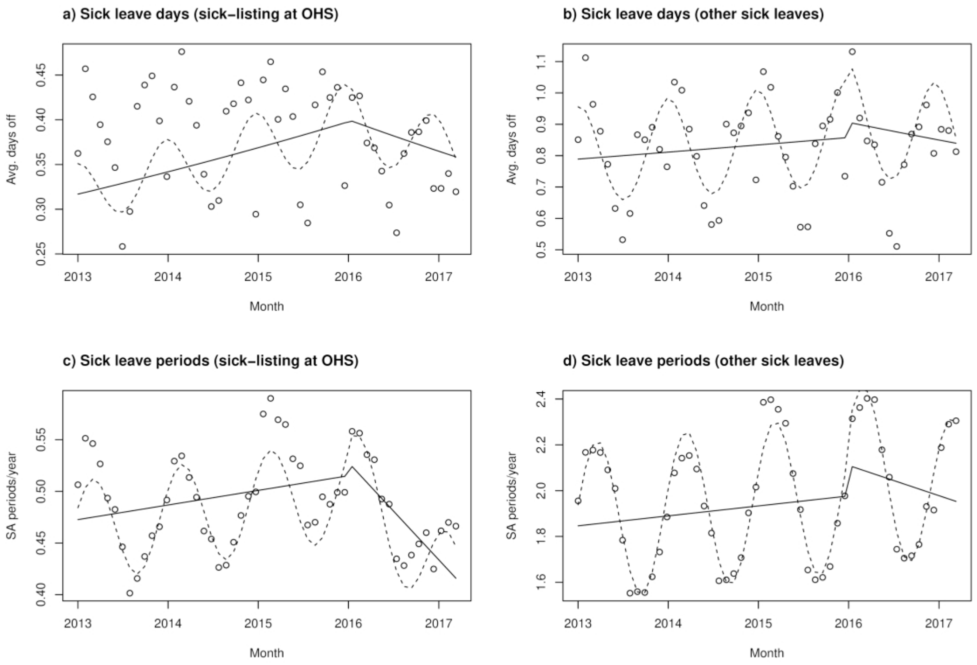

Figure 2: Model fits. Monthly averages of the number of all sick leave days (figures a) and b)) and periods (figures c) and d)). Observed data points denote to simple monthly averages and the lines represent GEE-model fits to employee-level data. The dashed line represents the full model prediction, including annual variations, whereas the solid line is the ITS-part of the model.
ITS=Interrupted time series analysis; GEE= generalised estimation equation

173x120mm (600 x 600 DPI)

Reporting checklist for cohort study.

Based on the STROBE cohort guidelines.

Instructions to authors

Complete this checklist by entering the page numbers from your manuscript where readers will find each of the items listed below.

Your article may not currently address all the items on the checklist. Please modify your text to include the missing information. If you are certain that an item does not apply, please write "n/a" and provide a short explanation.

Upload your completed checklist as an extra file when you submit to a journal.

In your methods section, say that you used the STROBE cohortreporting guidelines, and cite them as:

von Elm E, Altman DG, Egger M, Pocock SJ, Gotzsche PC, Vandenbroucke JP. The Strengthening the Reporting of Observational Studies in Epidemiology (STROBE) Statement: guidelines for reporting observational studies.

		Reporting Item	Page Number
**Title and abstract**			
Title	#1a	Indicate the study's design with a commonly used term in the title or the abstract	1

For peer review only

Abstract	#1b	Provide in the abstract an informative and balanced summary of what was done and what was found	2
**Introduction**			
Background / rationale	#2	Explain the scientific background and rationale for the investigation being reported	4
Objectives	#3	State specific objectives, including any prespecified hypotheses	5
**Methods**			
Study design	#4	Present key elements of study design early in the paper	5
Setting	#5	Describe the setting, locations, and relevant dates, including periods of recruitment, exposure, follow-up, and data collection	6
Eligibility criteria	#6a	Give the eligibility criteria, and the sources and methods of selection of participants. Describe methods of follow-up.	5
Eligibility criteria	#6b	For matched studies, give matching criteria and number of exposed and unexposed	n/a
Variables	#7	Clearly define all outcomes, exposures, predictors, potential confounders, and effect modifiers. Give diagnostic criteria, if applicable	9
Data sources / measurement	#8	For each variable of interest give sources of data and details of methods of assessment (measurement). Describe comparability of assessment methods if there is more than	5, 6, 9

For peer review only

		one group. Give information separately for for exposed and unexposed groups if applicable.	
Bias	#9	Describe any efforts to address potential sources of bias	10
Study size	#10	Explain how the study size was arrived at	5
Quantitative variables	#11	Explain how quantitative variables were handled in the analyses. If applicable, describe which groupings were chosen, and why	9
Statistical methods	#12a	Describe all statistical methods, including those used to control for confounding	10
Statistical methods	#12b	Describe any methods used to examine subgroups and interactions	10
Statistical methods	#12c	Explain how missing data were addressed	n/a
Statistical methods	#12d	If applicable, explain how loss to follow-up was addressed	n/a
Statistical methods	#12e	Describe any sensitivity analyses	12
**Results**			
Participants	#13a	Report numbers of individuals at each stage of study—eg numbers potentially eligible, examined for eligibility, confirmed eligible, included in the study, completing follow-	12

For peer review only

		up, and analysed. Give information separately for for exposed and unexposed groups if applicable.	
Participants	#13b	Give reasons for non-participation at each stage	n/a
Participants	#13c	Consider use of a flow diagram	n/a
Descriptive data	#14a	Give characteristics of study participants (eg demographic, clinical, social) and information on exposures and potential confounders. Give information separately for exposed and unexposed groups if applicable.	21
Descriptive data	#14b	Indicate number of participants with missing data for each variable of interest	n/a
Descriptive data	#14c	Summarise follow-up time (eg, average and total amount)	12
Outcome data	#15	Report numbers of outcome events or summary measures over time. Give information separately for exposed and unexposed groups if applicable.	22
Main results	#16a	Give unadjusted estimates and, if applicable, confounder-adjusted estimates and their precision (eg, 95% confidence interval). Make clear which confounders were adjusted for and why they were included	23
Main results	#16b	Report category boundaries when continuous variables were categorized	n/a
Main results	#16c	If relevant, consider translating estimates of relative risk into absolute risk for a meaningful time period	n/a

For peer review only

Other analyses	#17	Report other analyses done—e.g., analyses of subgroups and interactions, and sensitivity analyses	12, 23
**Discussion**			
Key results	#18	Summarise key results with reference to study objectives	16
Limitations	#19	Discuss limitations of the study, taking into account sources of potential bias or imprecision. Discuss both direction and magnitude of any potential bias.	18
Interpretation	#20	Give a cautious overall interpretation considering objectives, limitations, multiplicity of analyses, results from similar studies, and other relevant evidence.	17
Generalisability	#21	Discuss the generalisability (external validity) of the study results	17
**Other Information**			
Funding	#22	Give the source of funding and the role of the funders for the present study and, if applicable, for the original study on which the present article is based	16–19

The STROBE checklist is distributed under the terms of the Creative Commons Attribution License CC-BY. This checklist was completed on 12. November 2020 using https://www.goodreports.org/, a tool made by the EQUATOR Network in collaboration with Penelope.ai

For peer review only

BMJ Open

An intervention targeted at physicians’ treatment of musculoskeletal disorders and sickness certification: An interrupted time series analysis

Journal:	*BMJ Open*
Manuscript ID	bmjopen-2020-047018.R1
Article Type:	Original research
Date Submitted by the Author:	09-Jul-2021
Complete List of Authors:	Kausto, Johanna; Finnish Institute of Occupational Health<br>Rosenström, Tom Henrik; University of Helsinki Faculty of Medicine, Department of Psychology and Logopedics<br>Ervasti, Jenni; Finnish Institute of Occupational Health<br>Pietiläinen, Olli; University of Helsinki, Department of Public Health<br>Kaila-Kangas, Leena; Finnish Institute of Occupational Health<br>Rahkonen, Ossi; University of Helsinki, Department of Public Health<br>Harkko, Jaakko; University of Helsinki Faculty of Social Sciences<br>Vaananen, Ari; Finnish Institute of Occupational Health<br>Kouvonen, Anne; University of Helsinki, Faculty of Social Sciences; Queen's University Belfast, Administrative Data Research Centre<br>Lallukka, Tea; University of Helsinki, Department of Public Health
**Primary Subject Heading**:	Public health
Secondary Subject Heading:	Evidence based practice, Occupational and environmental medicine
Keywords:	OCCUPATIONAL & INDUSTRIAL MEDICINE, PUBLIC HEALTH, Musculoskeletal disorders < ORTHOPAEDIC & TRAUMA SURGERY

SCHOLARONE™
Manuscripts

I, the Submitting Author has the right to grant and does grant on behalf of all authors of the Work (as defined in the below author licence), an exclusive licence and/or a non-exclusive licence for contributions from authors who are: i) UK Crown employees; ii) where BMJ has agreed a CC-BY licence shall apply, and/or iii) in accordance with the terms applicable for US Federal Government officers or employees acting as part of their official duties; on a worldwide, perpetual, irrevocable, royalty-free basis to BMJ Publishing Group Ltd ("BMJ") its licensees and where the relevant Journal is co-owned by BMJ to the co-owners of the Journal, to publish the Work in this journal and any other BMJ products and to exploit all rights, as set out in our licence.

The Submitting Author accepts and understands that any supply made under these terms is made by BMJ to the Submitting Author unless you are acting as an employee on behalf of your employer or a postgraduate student of an affiliated institution which is paying any applicable article publishing charge ("APC") for Open Access articles. Where the Submitting Author wishes to make the Work available on an Open Access basis (and intends to pay the relevant APC), the terms of reuse of such Open Access shall be governed by a Creative Commons licence – details of these licences and which Creative Commons licence will apply to this Work are set out in our licence referred to above.

Other than as permitted in any relevant BMJ Author's Self Archiving Policies, I confirm this Work has not been accepted for publication elsewhere, is not being considered for publication elsewhere and does not duplicate material already published. I confirm all authors consent to publication of this Work and authorise the granting of this licence.

For peer review only

B M J O P E N
Public health research

An intervention targeted at physicians' treatment of musculoskeletal disorders and sickness certification: An interrupted time series analysis

Johanna Kausto[1], Tom Rosenström[1, 2], Jenni Ervasti[1], Olli Pietiläinen[3], Leena Kaila-Kangas[1], Ossi Rahkonen[3], Jaakko Harkko[4], Ari Väänänen[1], Anne Kouvonen[4,5], Tea Lallukka[3]

1 Finnish Institute of Occupational Health, Helsinki, Finland

2 Department of Psychology and Logopedics, Faculty of Medicine, University of Helsinki, Helsinki, Finland

3 Department of Public Health, University of Helsinki, Helsinki, Finland

4 Faculty of Social Sciences, University of Helsinki, Helsinki, Finland

5 Administrative Data Research Centre - Northern Ireland, Queen's University Belfast, Belfast, UK

*Corresponding author:

Johanna Kausto

Specialized Researcher

Finnish Institute of Occupational Health
PL 18
00032 TYÖTERVEYSLAITOS

FINLAND

johanna.kausto@ttl.fi

tel. + 358 30 474 2509, + 358 43 8200 698

Counts: Abstract (259), main text (4672), Tables (3), Figures (2)

Keywords: Sick leave [MeSH], Practice guidelines [MeSH], Occupational Health Service [MeSH], Musculoskeletal Diseases [MeSH]

For peer review only

Abstract

Objectives: An intervention was carried out at the occupational health care services (OHS) of the City of Helsinki beginning in 2016. We investigated the effects of the intervention on employee sick leave applying interrupted time-series analysis.

Design: Register-based cohort study with a quasi-experimental study design.

Setting: Employees of the City of Helsinki.

Participants: We analysed individual-level register-based data on all employees who were employed by the City for any length of time between 2013 and 2018 (a total 86,970 employees and 3,014,075 sick leave days). Sick leave days and periods that were OHS-based constituted the intervention time series and the rest of the sick leave days and periods contributed to the comparison time series.

Intervention: Provided recommendations for physicians on managing pain and prescribing sick leave for low back, shoulder, and elbow pain

Outcome measures: Number of sick leave days per month and sick leave periods per year.

Results: For all sick leave days prescribed in OHS, there was no immediate effect of the intervention, whereas a gradual intervention effect decreasing the number of OHS-based sick leave days was detected. On average, the intervention was estimated to have saved 2.5 sick leave days per year per employee. For other sick leave days, there was an immediate increase in the level of sick leave days after the intervention and a subsequent gradual effect of decreasing the number of sick leave days

Conclusions: The intervention reduced employee sick leaves and therefore it is likely that it had led to direct cost savings. However, further evidence for causal inferences is needed.

For peer review only - http://bmjopen.bmj.com/site/about/guidelines.xhtml

For peer review only

Article Summary

Strengths and limitations of this study

- This study presents novel information on the effects of introducing recommendations for management of pain and prescribing sick leave for musculoskeletal disorders, on employee sick leave.

- Comprehensive individual level data from employer registers were linked to individual level data from national registers.

- We applied interrupted time series analysis with a comparison time series which is a strong quasi-experimental design, recommended for examining population-level interventions.

-Intervention status was not obtainable for the entire follow-up time, and information on diagnoses was available only for sick leave periods that lasted over 11 calendar days.

For peer review only

Introduction

Musculoskeletal disorders (MSD) are highly prevalent among working age population, and they result in extensive disability costs both for the individuals and the society. In Finland in 2019, 27% of the days of sick leave periods lasting at least 11 calendar days were due to MSD [1] . Thus, sick leave is typically granted for musculoskeletal pain symptoms, such a s back, shoulder or elbow pain. However, scientific evidence does not support the beneficiality of long sick leaves. For example, it has been shown that when having low back pain, from a point of view of recovery, staying active is better than bed rest [2, 3].

Primary care doctors' advice can have a major impact on whether an individual takes sick leave and for how long [4]. In a follow-up study it was found that visiting a medical specialist because of a musculoskeletal disorder was associated with a delayed full return to work irrespective of the severity of ailment [5]. Furthermore, earlier studies have found that sick listing (sick leave prescribing) practices vary a lot across physicians [6, 7]. A Finnish study showed that the differences in the length of prescribed sick leaves cab be up to 8-fold [8]. In line with this, a survey found that Finnish physicians experienced that they needed more information, guidance and training on sick listing [9].

Against this background, an intervention was carried out at Occupational Health Helsinki, which provides occupational health care services (OHS) for the City of Helsinki employees. The intervention was launched in 2016. Introducing and increasing the use of shared guidelines on the management of pain and work disability and prescribing sick leave for low

For peer review only - http://bmjopen.bmj.com/site/about/guidelines.xhtml
For peer review only

back, shoulder and elbow pain was the central element in the intervention. To investigate the effects of this intervention on employee sick leaves, we carried out prospective controlled time series and binomial regression analyses using register-based data.

Methods

Study design and population

Data were collected as a part of the Helsinki Health Study [10]. The study population includes a sample of the employees of the City of Helsinki. Women represent 70% of the employees who work in general local administration, social and health care, education and culture, public transport, and technical services. All employees share the same personnel administration, policies, and occupational health care services [10]. Employees can use the services (including primary care services for other than work-related conditions) of their OHS provider, Occupational Health Helsinki; or they can seek treatment from public or private health care. The employees of the City of Helsinki can use self-certification for sick leaves lasting up to five days. In the case of self-certification, a medical certificate is not needed, but the employee notifies the supervisor when taken ill. In 2016, self-certification was mostly used among young employees (under 30 years of age) [11].

For the purposes of this study, individual-level register-based data of all the employees who were employed by the City for any length of time between 2013 and 2018 (n= 86,970) were analysed. Background information on participants were drawn from employer's registers. Information on all sick leave periods (start and end dates and diagnostic codes (ICD-10)) during this time were derived from employer's registers and the national sickness insurance

For peer review only - http://bmjopen.bmj.com/site/about/guidelines.xhtml

register of the Social Insurance Institution of Finland (SII). ICD-10 codes for sick leave periods were available from the SII for sick leave periods lasting more than 11 calendar days. Data on chronic somatic and psychiatric illnesses and the purchases of prescribed psychotropic medication were obtained from the Register of Special Reimbursement for Medication Purchases (SII). Data from these three registers were linked based on unique individual numbers of the participants. Such numbers are assigned to each citizen of Finland at birth and for migrants when they get a residence permit, and they are common to all administrative registers, enabling extensive record linkages [12]. The study follows the Helsinki Health Study (HHS) protocol in line with the University of Helsinki's guidelines and the EU and Finnish data legislation. The ethics committees of the Department of Public Health, the University of Helsinki and the health authorities of the City of Helsinki have approved the HHS study. The City of Helsinki and register holders have given permissions for data linkage.

Patient and Public Involvement

No patients involved.

Study context

Occupational Health Care in Finland

In the Finnish occupational health care service (OHS) system all employees are entitled to statutory preventive OHS provided by the employer. Most employers offer state-subsidised free primary care services for their employees on top of the statutory preventive OHS. OHS providers in Finland provide primary care services to employees in a way that is comparable

For peer review only - http://bmjopen.bmj.com/site/about/guidelines.xhtml

to primary care in international comparisons in substance, but differs in that the service is targeted only to the working population [13]. The statutory tasks of OHS include assessment of the health and safety aspects of the work, assessment and monitoring of employees' health and work ability, making initiatives for improvement and monitoring their implementation, advice and guidance, monitoring employees with disabilities and referring them to rehabilitation, co-operation with representatives of other health care services and social insurance, participation in organising first aid at the workplace, participation in activities that maintain work ability, and monitoring the quality and impact of occupational health care activities [14].

Intervention

Occupational Health Helsinki provides occupational health care to the almost 40,000 employees working annually in the City departments. The employees represent hundreds of different occupations, and a rather large supply of health care services supporting the employees' work ability and the functioning of work units is offered. The Occupational Health Helsinki staff consists of 150 health care professionals.

The aims of the intervention were to increase the quality and effects of occupational health care, to increase the use of mutually created and shared guidelines based on available scientific evidence in assessing work disability, to provide a practical instrument for physicians in managing pain and prescribing sick leave in the form of recommendations, to help physicians to take into account the complexity of the association between pain and work disability, and to provide up-to-date information to the patient about their pain

For peer review only - http://bmjopen.bmj.com/site/about/guidelines.xhtml

condition, its treatment and how it may affect work and work ability. The goal was to reduce employee sickness absence and improve work ability.

Recommendations on work disability management and prescribing sick leave for low back pain, shoulder pain and elbow pain were launched first in January-2016 and they were the central and the most noticeable component in the intervention. Recommendations were launched in a form of three guideline papers (one for each diagnostic category). Training regarding the implementation of the guidelines was organised in OHS for physicians and all other relevant health care professionals such as physiotherapists and nurses. Short educational sessions were offered, and an e-learning course and some coaching sessions were led by a pain specialist. An intensive follow-up of the sick leave trends was started in OHS. For instance, recent trends of sick leave were reported in the monthly meetings for the physicians. All new relevant OHS employees were briefed about the guidelines.

As an example, in the case of low back pain, the recommendation (Appendix 1) emphasised that the condition is very common and nearly all people experience a disabling low back pain (i.e. pain, ache and stiffness in the area of lower back) at some point in their lives. The condition is usually benign in nature, heals spontaneously in a few days, and 90% of the patients will fully recover in 4 to 6 weeks. Still, possible serious causes for back pain should be ruled out. In sick listing the following points should be considered: In many cases sick leave is not needed; There is no indication that physically strenuous work would slow down recovery; If sick listing is necessary, a few days up to a week should be sufficient in light duties; If work includes heavy lifting, bending or rotation, two weeks of full time sick leave may be needed; Factors that provoke pain should be temporarily modified; Factors that suggest an increased risk for prolonged back pain and work disability include sleeping

For peer review only - http://bmjopen.bmj.com/site/about/guidelines.xhtml

problems, depression and anxiety, fear avoidance beliefs, multisite pain, and problems at the workplace and dissatisfaction with work. The recommendations included a checklist for the physicians to be used at the appointment.

Intervention status

Sick leave periods that were OHS-based (if a sick leave was preceded by a likely related appointment at OHS within 11 days, it was approximated that sick-listing had taken place at OHS) constituted the intervention time series and the rest of the sick leave periods (no appointment at OHS, but appointment and sick-listing had taken place elsewhere or the employee had used self-certification, in which case there had been no medical appointment at all) contributed to the comparison time series. The available registers contained information of appointments in the OHS until 20th April 2017.

Outcome

We calculated the number of sick leave days per month and sick leave periods per year.

Covariates

Covariates that were available in the registers and were regarded as potential confounders included age, sex, occupational class (based on occupational title and categorised as upper grade non-manual employees, intermediate grade non-manual employees, lower grade non-manual employees and manual workers), job contract (permanent, temporary) valid 1st January 2016, chronic somatic illnesses (derived from the Register of Special

For peer review only - http://bmjopen.bmj.com/site/about/guidelines.xhtml

Reimbursement for Medication Purchases, and included diabetes, heart disease, rheumatoid arthritis, chronic asthma, stage 2 hypertension, Parkinson's disease, epilepsy, uremia, bowel disease, multiple sclerosis disease, and diseases of pancreas and categorized as 0=no, 1=yes) valid 1st January 2016, and purchases of prescribed psychotropic medication (Anatomic Therapeutic Chemical, ATC, classification N06 or N05 categorised as 0=no, 1=yes) between 1st November 2015 and 1st November 2016.

Statistical analyses

We used an interrupted time series (ITS) design with a comparison time series [15-17]. The observational unit in the analyses was a sick leave day or period. The ITS analysis uses time-series (a continuous sequence of observations on a population-level taken repeatedly at equal intervals over time) for a specific outcome to establish an underlying trend which is "interrupted" by an intervention at a specific point of time. A strength of ITS analyses is that they are generally not affected by typical confounding factors (such as age distribution or socioeconomic status) as they model a time trend in a context where the population composition (in terms of the confounding factors) remains rather constant before vs. after the intervention. Measured time varying confounders can be controlled for in the regression models. Unmeasured or unknown time varying confounders can be controlled by adding a comparison group (not exposed to the intervention) [15]. It has been recommended that the covariate balance between the intervention and comparison time series is explored, though it is not a prerequisite for the analysis [16].

Our integer-valued (count, or proportion) outcome variable was days of sick leave per month (range 0–30, or from 0 to days exposed within the 30-day period, if not with the

For peer review only - http://bmjopen.bmj.com/site/about/guidelines.xhtml

employer for the full 30-day period), recorded separately for each month. Although each employee was followed up for multiple months, different employees may have different time average rate of sick leave days, introducing statistical dependencies to the observations. As our interests pertained to a population-level causal effect, within employee clustering in the outcome was considered a nuisance variable and its effects were modeled using generalised estimating equations [18]. Given the data characteristics above, the outcome was modeled using generalised (binomial) linear regression model [19] and the models were estimated with generalised estimating equations. Although our data was in the form of monthly counts, an exponentiated binomial regression coefficient can be interpreted as an odds ratio (OR) for a sick-leave day (vs. no sick leave) for a randomly chosen day, adjusting for the other covariates in the model. As a sensitivity analysis, we also tested quasi-binomial generalised linear model with usual maximum likelihood estimation, which gave similar results with narrower confidence intervals.

The main test of our hypothesis pertained to the model equation

$$g^{-1}(Y) = \beta_0 + \beta_{slope}(m - m_0) + \beta_{immediate}X + \beta_{gradual}X(m - m_0),$$

where Y is the outcome, g the logit-link function, X is 1 for all months after the intervention launch date (1st of January 2016) and 0 before it, and $(m - m_0)$ is time (months, centered around the time of intervention [20] , m_0). Here, β_{slope} is the linear trend effect before the intervention, whereas $\beta_{slope} + \beta_{gradual}$ is the trend after the intervention took place. The intercept before the intervention is β_0 and that after the intervention $\beta_0 + \beta_{immediate}$. Thus, $\beta_{immediate}$ is the immediate intervention effect and $\beta_{gradual}$ the gradual intervention effect that accumulates at the rate $\beta_{gradual}$ per month after the intervention (for the remaining observation period). Besides this usual ITS setting, we took into account the annual

For peer review only - http://bmjopen.bmj.com/site/about/guidelines.xhtml

fluctuation of sick leave by modelling an annual trend estimating coefficients for two additional covariates with values $\sin(m2\pi/12)$ and $\cos(m2\pi/12)$, where the factor $2\pi/12$ scales monthly data points m to the annual cycles [21] .

Because our outcome data timepoints (months) could include sick leave days from prescribed at OHS and other sources, defining a covariate for intervention group membership would have required us to prioritise either OHS or other sources. It is typical in CITS design to include a binary covariate that gets a value 1 when a unit (here a person) belongs to the intervention group and value 0 when in the control group. In this study, participants had sick leaves both from OHS and non-OHS sources. Instead of formal testing of such covariate, we conducted a separate ITS-design and analyses for the intervention and comparison groups. Both crude models and models adjusted for the covariates were fitted. Analyses were run separately for men and women, for short (<= 11 calendar days) and longer (> 11 calendar days) sick leave periods, and for sick leave periods (>=11 calendar days) in the diagnostic categories of back pain (ICD-10 M54.5), shoulder pain (ICD-10 M75) and elbow pain (ICD-10 M77.1).

As the sick leave recommendations potentially had an effect both on the number of sick leave days and periods, the number of consecutive sick leave periods were computed within index month plus 5 immediately preceding months. Monthly sample averages of numbers of periods were investigated with otherwise similar linear models as in above but using a six-month moving-average error structure. The “arima” function of R version 3.5.1 (2018-07-02) was used. Local regression lines for figures were drawn using loess-function of “stats” R package, version 3.5.1, with the default options. The same software was used for all the analyses.

For peer review only - http://bmjopen.bmj.com/site/about/guidelines.xhtml

Results

Descriptive results

Our data included information on sick leaves of a total of 86,970 employees covering more than 73 months. Due to workforce turnover, the monthly sample size of employees averaged at 41,289, with a minor variance across months (min 37,567, max 45,520; SD 2,054). Overall, 70% of all employees were on sick leave at least once during the follow-up and 33% of the employees were prescribed sick leave at OHS at least once between 1st January 2013 and 21st April 2017. Of those employees who were prescribed sick leave at OHS, 95% were prescribed sick leave also elsewhere (or they used self-certification). Of those who were prescribed sick leave elsewhere (or used self-certification) at least once, 46% were prescribed sick leave also at OHS. Of all employees, 30% were not on sick leave at all. On average, employees were prescribed 0.36 days of sick leave per month at OHS and 0.82 sick leave days per month were prescribed elsewhere or were self-certified. The respective mean numbers of sick leave periods per employee per year were 0.49 and 1.96.

Descriptive statistics for all employees, employees with sick leaves prescribed at OHS and employees with other sick leaves (prescribed elsewhere or self-certificated) are shown in Table 1 and Table 2. Those employees who were prescribed sick leave at OHS at least once by 21st April 2017, were more often permanently employed, older, female, and intermediate or lower level non-manual workers (Table 1).

Monthly averages of the number of all sick leave days

For peer review only - http://bmjopen.bmj.com/site/about/guidelines.xhtml

Since it is generally recommended to draw simple plots of ITS analyses before entering into more complex model fitting [15, 16], we started by examining the monthly average days of sick leave per employee. Based on a visual inspection of monthly averages, the number of all sick leave days prescribed at OHS declined shortly after the intervention had started in 2016 (Figure 1a). and similar temporal changes were not observed for other sick leave periods (Figure 1b). After 21st April 2017, our register data could not differentiate between sick leave days that were OHS-based and other sick leave days, but we had a longer stretch of data when counting in all sick leaves. A decline in sickness absence after intervention was seen in a visual inspection of all sick leaves (thick line for local-regression smoother), as well as annual periodic variation (thin dashed line in Figure 1c). When removing cyclic annual variation and a linear trend, the visual suggestion of an intervention effect remained. However, sick leaves appeared to return to preceding rates towards the end of the longest available follow-up data (in 2018) (Figure 1d). It should be noted that Panels in Figures 1 and 2 are not comparable to each other as Y-axis are different. The figures illustrate how the trend in sick leave days changes at the time of the intervention rather than compare absolute values. Due to relatively small change in trend, similar Y-axis would not illustrate the trend.

Formal modeling (adjusting for seasonal variation) did not reveal an immediate intervention effect ($\beta_{immediate}$ = 0.01, 95% CI = (-0.052–0.072)), but did detect a gradual intervention effect ($\beta_{gradual}$ = -0.007, 95% CI = (-0.013– -0.001)) on the number of OHS-based sick leave days. The corresponding numbers were similar in direction but not statistically significant in the comparison group ($\beta_{immediate}$ = 0.068, 95% CI = (-0.065–0.201)), and ($\beta_{gradual}$ = -0.009, 95% CI = (-0.022– 0.004)) (data not shown). These formal findings would support a gradual

For peer review only - http://bmjopen.bmj.com/site/about/guidelines.xhtml

effect of intervention, but we were able to achieve a greater statistical power by accessing the employee-level data instead of the above-discussed monthly averages.

Results on employee-level data: The number of all sick leave days

We modelled the number of sick leave days using binomial regression on monthly sick leave days per employee. Figures 2 a and b illustrate the model predictions for the number of all sick leave days. For convenient illustration, we plotted the model estimates overlaid on the above discussed monthly averages, but importantly these models use 2,160,445 observations from 75,962 individuals instead of 52 monthly averages. The exact binomial regression coefficients (with logit-link function) as well as odds ratios for a sick leave day at index month vs. preceding month are presented in Table 3.

We observed a clear gradual diminishing trend in the number of all-cause OHS-based sick leave days (first model, Table 3). A similar gradual trend was found in the comparison group (sick-listing elsewhere or self-certification), but that was offset by an increase in the level of the number of sick leave days at the time of intervention. Adjusting for the covariates (Table 3, model 2) did not affect the estimates significantly. We additionally ran the analyses separately for men and women (not shown in the table). The results were in line with the first model (total sample). The intervention effect found in all-cause sick leave was not paralleled in diagnostic group-specific findings (models 4 to 6 in Table 3).

While a gradual intervention effect of odds ratio 0.986 may appear small, we can illustrate the effect of the intervention in a practical way as follows. Assuming employees on average have 1.2 sick-leave days per month (cf. Fig. 1c), their baseline risk rate per day would be 0.04. Small risk ratios closely match odds ratios. Thus, if the other factors have already been

For peer review only - http://bmjopen.bmj.com/site/about/guidelines.xhtml

adjusted for, the intercept for a logistic model would be $\beta_0 = \log(0.04)$. Then the gradual intervention effect of $OR_{gradual} = 0.986$ would imply that odds ratio of a sick-leave day one year after the intervention has reduced to $\exp(\beta_0 + 12 \times \log(OR_{gradual})) \approx 0.034$ and the risk rate of a sick-leave day to approximately 0.033. Thus, the gradual intervention effect on average amounts to $(0.04 - 0.033) \times 30 \times 12 = 2.52$ avoided sick-leave days per year per employee. Expressed in another way, the intervention may have saved 2,520 sick-leave days per year per 1,000 employees.

Results on the employee-level data: The number of all sick leave periods

As a sensitivity analysis, we investigated the number of all-cause sick leave periods as an outcome. A sick leave period is a consecutive spell of sick leave days. We report results in units of periods per year per employee, although we computed periods per index month plus five preceding months (Figure 2 c and d). We analysed the monthly averages with a moving-average regression model. For all sick leave periods prescribed in OHS, we did not detect any immediate effect of intervention ($\beta_{immediate}$ = 0.017, 95% CI = (-0.011– 0.046)), but a gradual intervention effect diminishing the number of sick leave periods was found ($\beta_{gradual}$ = -0.009, 95% CI = (-0.013– -0.005)). The findings on the gradual effect were similar in the comparison group ($\beta_{gradual}$ = -0.014, 95% CI = (-0.026– -0.003)), whereas the immediate effect indicated that an increase of the sick leave periods occurred in the comparison group at the time of intervention ($\beta_{immediate}$ = 0.141, 95% CI = (0.067– 0.214)).

For peer review only - http://bmjopen.bmj.com/site/about/guidelines.xhtml

Discussion

In this register-based study among municipal employees, we analysed sick leave trends before and after an intervention targeted at physicians' management of musculoskeletal pain-related work disability and prescribing sick leave. The intervention was carried out in OHS setting. We examined monthly averages of all-cause sick leave days and found no immediate change of trend among OHS-based sick leave days but did detect a gradual change of trend decreasing the number of OHS-based sick leave days by 2.5 days per year per employee. The corresponding findings were similar in direction but not statistically significant for the comparison time series (sick-listing elsewhere or self-certification). In the employee-level data (with a larger statistical power), we found a clear gradual change of trend decreasing the number of OHS-based all-cause sick leave days. Similar gradual decreasing trend was found for the comparison time series, but that was offset by an increase in the level of sick leave days and periods at the time of intervention. A hypothetical company with 40,000 employees and a direct cost 250 of EUR per sick-leave day (numbers similar to those of the City of Helsinki)[22], would have saved $2520 \times 40 \times 250$ EUR, amounting to approximately 25 million EUR per year. If one assumes that the trend in OHS sick leaves would have followed changes in the comparison group instead of the pre-intervention trend, one could subtract the gradual effect in comparison group from that of the OHS group to derive a conservative OR estimate (0.994 = exp(-0.014+0.008), from Table 3 coefficients) and then repeat the calculation. This would yield 1.01 avoided sick-leave days per year per employee, with estimated approximate savings of 10.1 million EUR per year.

For peer review only - http://bmjopen.bmj.com/site/about/guidelines.xhtml

The knowledge base on physicians' sick-listing practices is accumulating. The present study suggests that the intervention affected the sick-listing practices of physicians working in Occupational Health Helsinki. There was a downward trend in the number of OHS-based sick leave days and periods. Nevertheless, the findings may also suggest that soon after the intervention carried out in OHS, the employees increasingly sought treatment elsewhere (or used self-certification increasingly) as a rise in the level of other sick leaves was detected. This trend leveled down, however.

As far as we know, there are no previous studies investigating the effects of introducing guidelines on prescribing sick leaves (as compared to the situation where no guidelines exist). There are a few studies though that compare different ways of implementation to each other [23, 24] There are a great number of previous studies on interventions attempting to change physicians' behaviour in some other areas of clinical practice. Findings show that success in changing the behaviour and adherence to different clinical guidelines varies. Some studies report success in behaviour change [25, 26] whereas other studies report limited success or found interventions ineffective [27-29]. Moreover, a change in physician's behaviour does not automatically reflect in patient outcomes [28]. Guidelines have been found to be a necessary but an insufficient step in changing clinical care [30]. The importance of proactive implementation of guidelines and efforts to include the new practice into existing organisational procedures, which both were attempted in this intervention, have been emphasised to achieve sustainable results [31, 32]. As the context of the intervention is important, the results of an intervention in one environment cannot directly be applied to another context.

For peer review only

A strength of this study is its design based on register data on all employees. We could link comprehensive data from employer registers to data from national registers using personal identification numbers. The use of administrative registers in research always entails limitations as they are not typically collected for research purposes. Nevertheless, in general the quality of registers in Finland as in other Nordic countries is good and administrative registers offer unique opportunities and are frequently used in research.

Applied ITS analysis with a comparison time series has been recommended for analysing population-level interventions [15, 33]. A limitation was that the intervention status was approximated (an OHS-based sick leave was preceded by a likely relevant appointment at OHS) and the information was not available for the entire follow up period. The validity of the comparison group can be debated [17] as all employees could be prescribed sick leave both at OHS and elsewhere (or use self-certification in short sick leaves). However, only the OHS professionals were exposed to the intervention and the time series for the intervention and comparison groups were analysed separately. Thus, our analysis should be thought of as being in between ITS and CITS in what comes to scientific value of evidence.

Because the intervention was targeted at managing pain and prescribing sick leave from the first day of work disability, a limitation was that we had access to diagnostic data only for sick leave periods that lasted over 11 calendar days. This, together with the low base rate for specific diagnoses, may explain the result that the intervention effect found in among

For peer review only - http://bmjopen.bmj.com/site/about/guidelines.xhtml

all-cause sick leaves was not paralleled in diagnostic group-specific findings of sick leaves (lasting over 11 calendar days) due to low back, shoulder, and elbow pain. Musculoskeletal disorders constitute a major cause for sick leave. The fact that an intervention effect was seen among all-cause sick leaves (regardless of the length of the sick leave period) may also indicate that recommendations given for these three specific disorders influenced how the physicians managed work disability and prescribed sick leave altogether. Unfortunately, we did not have information on part time sick leaves and for that reason we could not take this into account in the analyses. A sick leave day was counted as a sick leave day. In Finland, partial sickness benefit can be granted only after 11 days of full-time sickness absence. Partial sickness benefit days comprised only 7% of all compensated sickness benefit days in Finland in 2016.

The recommendations for physicians constituted chronologically the first and the main element in the intervention. Direct access to physiotherapists without a doctor's referral was introduced in the following year, and thereafter it was not possible to differentiate the effects of these different elements.

A gradual change of trend at the time of intervention was observed, resulting in a decline of the number of sick leave days and periods prescribed in OHS. This is consistent with the intervention being effective, but the causality of the relationship is unclear due to similar findings for the comparison group. The estimated reduction of 2.5 sick leave days per year per employee could leave to savings of 25 million euros for a large employer such as the City of Helsinki. This can be considered as an economically relevant intervention effect, but further evidence for its causal interpretation is needed.

For peer review only - http://bmjopen.bmj.com/site/about/guidelines.xhtml

For peer review only

Figure legends

Figure 1: Model fits. Monthly averages of the number of all sick leave days. Intervention status could be specified until 21st April 2017 (Figures a and b). In Figures c and d the intervention status is not differentiated. Vertical dashed lines indicate the time point of intervention (all figures). Circles denote to observed averages, thick lines to their local regression fits (smoothed averages) (all figures), and thin dashed line to annual periodic variation (Figure c).

Figure 2: Model fits. Monthly averages of the number of all sick leave days (figures a and b) and periods (figures c and d). Observed data points denote to simple monthly averages and the lines represent GEE-model fits to employee-level data. The dashed line represents the full model prediction, including annual variations, whereas the solid line is the ITS-part of the model.

ITS=Interrupted time series analysis; GEE= generalised estimation equation

For peer review only

For peer review only - http://bmjopen.bmj.com/site/about/guidelines.xhtml

Aknowledgements: We would like to thank MD, Chief Medical Officer Eevamaija Tuovinen, MD, PhD Helena Miranda, and CEO, PhD Tiina Pohjonen, Occupational Health Helsinki for providing information of the intervention and its context.

Conflicts of Interest: The authors have no conflicts of interest to declare. The funders had no role in the design of the study; in the collection, analyses, or interpretation of data; in the writing of the manuscript, or in the decision to publish the results.

Funding: This work was supported by the Social Insurance Institution of Finland (grant # 9/26/2019 JK, TR, JE, AV and LK-K), The Finnish Work Environment Fund (grant #190081 JK, TR, JE, AV and LK-K), the Academy of Finland (grants #1294514 and #319200 OR and TL) and the Economic and Social Research Council (ESRC) (grant # ES/S00744X/1 AK).

Open access funding provided by the Finnish Institute of Occupational Health.

Author Contributions: Conceptualisation and methodology: JK, TR, OP, AV, EJ, OR, JH, AK, and TL; writing–original draft preparation: JK and TR; writing–review and editing: JK, TR, EJ, OP, LKK, OR, JH, AV, AK, TL; project administration: JK.

All authors have read and agreed to the published version of the manuscript.

"We used the **STROBE cohort checklist** when writing our report “An intervention targeted at physicians’ treatment of musculoskeletal disorders and sickness certification: Aninterrupted time series analysis”.

Ethics approval and consent to participate: The ethics committees of the Department of Public Health, the University of Helsinki and the health authorities of the City of Helsinki approved the Helsinki Health Study protocol. The ethical approval applies to the current study. The participants gave written informed consent to participate in the study.

Provenance and peer review: Not commissioned; externally peer reviewed.

Data sharing statement: No additional data are available.

For peer review only

Table 1. Descriptive statistics for all employees, employees with sick leaves prescribed at OHS[1] and employees with sick leave prescribed elsewhere or self-certified (other sick leaves)*.

Characteristic	Sick-listing at OHS (n= 25200)	Other sick leaves (n=52174)	All (n=75962)
Age (Mean, SD)	44.67 (12.32)	42.16 (13.02)	40.27 (14.08)
Men (%)	20	23	27
Occupational class (%)			
Upper grade non-manual employees	21	24	27
Intermediate grade non-manual employees	27	25	22
Lower grade non-manual employees	37	36	34
Manual workers	16	15	17
Job contract (%)			
Permanent	82	67	54
Prescribed reimbursed purchases of medication for chronic diseases (%)			
Yes	21	17	16
Prescribed reimbursed psychotropic medication (%)			
Yes	12	9	8

* Note that many employees were prescribed sick leave both at OHS and elsewhere (or used self-certification). The group "All" contains also employees with no sick leaves.

OHS=Occupational health care services.

For peer review only

For peer review only - http://bmjopen.bmj.com/site/about/guidelines.xhtml

Table 2. Monthly average numbers of sick leave days per month per employee by intervention status (years 2013–2017).

	Sick-listing at OHS[1]			Other sick leaves		
	Average	Range	SD	Average	Range	SD
All sick leave days	0.380	0–30	2.59	0.840	0–30	3.40
All short sick leave periods[a]	0.117	0–25	0.79	0.392	0–24	1.24
Sick leave for low back pain[b]	0.005	0–30	0.36	0.007	0–30	0.39
Sick leave for shoulder pain[b]	0.020	0–30	0.71	0.014	0–30	0.59
Sick leave for elbow pain[b]	0.004	0–30	0.28	0.002	0–30	0.19

OHS=Occupational health care services

[a] l<= 11 calendar days, [b] > 11 calendar days

For peer review only

For peer review only - http://bmjopen.bmj.com/site/about/guidelines.xhtml

Table 3. Binomial regression models predicting the number of sick leave days.

	Sick listing at OHS				Other sick leaves			
	Coefficient β	Std. error	OR[1)]	95% CI	Coefficient β	Std. error	OR[1)]	95% CI
Sick leave days (all periods, all DC:s) (Crude model)[2)] (n=2 160 445 employee-months, n=75,962 employees)								
Immediate intervention effect	0.012	0.035	1.012	0.95–1.08	0.060**	0.018	1.062	1.03–1.10
Gradual intervention effect	-0.014***	0.003	0.986	0.98–0.99	-0.008***	0.002	0.992	0.99–1.00
Linear trend before intervention (Slope)	0.006***	0.001	1.006	1.00–1.01	0.002***	0.001	1.002	1.00–1.00
Sick leave days (all periods, all DC:s) (Fully adjusted model)[2)] (n=2 148 566 employee-months, n= 74,701 employees)								
Immediate intervention effect	-0.003	0.032	0.997	0.94–1.06	0.057**	0.018	1.059	1.02–1.10
Gradual intervention effect	-0.010**	0.003	0.990	0.98–1.00	-0.007***	0.002	0.993	0.99–0.99
Linear trend before intervention (Slope)	0.007***	0.001	1.007	1.01–1.01	0.003***	0.001	1.003	1.00–1.01
Sick leave days (short periods, < 11 calendar days, all dg:s) [2)] (n=2 160 445 employee-months, n=75,962 employees)								
Immediate intervention effect	0.105***	0.024	1.111	1.06–1.16	0.196***	0.010	1.217	1.19–1.24
Gradual intervention effect	-0.020***	0.002	0.980	0.98–0.98	-0.009***	0.001	0.991	0.99–0.99
Linear trend before intervention (Slope)	0.001	0.001	1.001	1.00–1.00	-0.003***	<0.001	0.997	1.00–1.00
Sick leave days in low back pain (periods >= 11 calendar days) [3)] (n=2 160 445 employee-months, n=75,962 employees)								
Immediate intervention effect	-0.547	0.340	0.579	0.30–1.13	-0.205	0.283	0.815	0.47–1.42
Gradual intervention effect	0.040	0.035	1.041	0.97–1.12	-0.019	0.031	0.981	0.92–1.04
Linear trend before intervention (Slope)	-0.004	0.009	0.996	0.98–1.01	-0.004	0.007	0.996	0.98–1.01

OHS=Occupational health care services, DC=diagnostic code. OR=Odds ratio, CI=Confidence interval. *p < 0.05, **p < 0.01, *** p< 0.001.

[1)] Immediate intervention effect: Odds ratio was estimated for any employee to have any sick leave day after vs. before the intervention. Gradual intervention effect: Odds ratio was estimated for any employee to have a sick leave day at index month vs. preceding month during the period after the intervention.[2)] Follow-up from 1st January 2013 to 21 st April 2017 [3)] Follow-up from 1st January 2013 to 31st December 2019. Adjusted for age, sex, occupational class, job contract, prescribed purchases of medication for chronic diseases, prescribed psychotropic medication.

For peer review only - http://bmjopen.bmj.com/site/about/guidelines.xhtml

Table 3. Binomial regression models predicting the number of sick leave days (Continued).

	Sick-listing at OHS				Other sick leaves			
	Coefficient β	Std. error	OR[1)]	95% CI	Coefficient β	Std. error	OR[1)]	95% CI
Sick leave days for shoulder pain (periods >= 11 calendar days)[3)] (n=2 160 445 employee-months, n=75,962 employees)								
Immediate intervention effect	0.365	0.207	1.441	0.96–2.16	-0.426	0.292	0.653	0.34–1.16
Gradual intervention effect	-0.023	0.023	0.977	0.93–1.02	0.013	0.025	1.013	0.97–1.06
Linear trend before intervention (Slope)	-0.004	0.008	0.996	0.98–1.01	-0.003	0.008	0.997	0.98–1.01
Sick leave days for elbow pain (periods >= 11 calendar days)[3)] (n=2 160 445 employee-months, n=75,962 employees)								
β immediate	-0.964	0.514	0.381	0.14–1.04	-0.398	0.486	0.672	0.26–1.74
β gradual	-0.034	0.072	0.967	0.84–1.11	-0.079	0.084	0.924	0.78–1.09
Slope	0.003	0.014	1.003	0.98–1.03	-0.001	0.018	0.999	0.96–1.04

OHS=Occupational health care services, DC=diagnostic code. OR=Odds ratio, CI=Confidence interval. *p < 0.05, **p < 0.01, *** p< 0.001.

[1)] Immediate intervention effect: Odds ratio was estimated for any employee to have any sick leave day after vs. before the intervention. Gradual intervention effect: Odds ratio was estimated for any employee to have a sick leave day at index month vs. preceding month during the period after the intervention.[2)] Follow-up from 1st January 2013 to 21 st April 2017 [3)] Follow-up from 1st January 2013 to 31st December 2019. Adjusted for age, sex, occupational class, job contract, prescribed reimbursed purchases of medication for chronic diseases, prescribed reimbursed psychotropic medication.

For peer review only - http://bmjopen.bmj.com/site/about/guidelines.xhtml

References

1. *Tilastotietokanta Kelasto (Statistics in Finnish)*. [cited 2020 20.10.]; Available from: https://www.kela.fi/kelasto.
2. van Tulder, M., et al., *Chapter 3. European guidelines for the management of acute nonspecific low back pain in primary care.* Eur Spine J, 2006. **15 Suppl 2**: p. S169-91.
3. Costa-Black, K.M., et al., *Back pain and work.* Best Pract Res Clin Rheumatol, 2010. **24**(2): p. 227-40.
4. Black, C.M., *Sickness absence and musculoskeletal disorders.* Rheumatology (Oxford), 2012. **51**(2): p. 204-5.
5. Lotters, F.J., M. Foets, and A. Burdorf, *Work and health, a blind spot in curative healthcare? A pilot study.* J Occup Rehabil, 2011. **21**(3): p. 304-12.
6. Englund, L., G. Tibblin, and K. Svardsudd, *Variations in sick-listing practice among male and female physicians of different specialities based on case vignettes.* Scand J Prim Health Care, 2000. **18**(1): p. 48-52.
7. Arrelov, B.E., L. Borgquist, and K.F. Svardsudd, *Influence of local structural factors on physicians' sick-listing practice: a population-based study.* Eur J Public Health, 2005. **15**(5): p. 470-4.
8. Kankaanpaa, A.T., J.K. Franck, and R.J. Tuominen, *Variations in primary care physicians' sick leave prescribing practices.* Eur J Public Health, 2012. **22**(1): p. 92-6.
9. Hinkka, K., et al., *Physicians' experiences with sickness absence certification in Finland.* Scand J Public Health, 2019. **47**(8): p. 859-866.
10. Lahelma, E., et al., *Cohort profile: the Helsinki Health Study.* Int J Epidemiol, 2013. **42**(3): p. 722-30.
11. Sumanen, H., et al., *Age-group differences in sickness absence among the employees of the City of Helsinki 2002–2016 (Article in Finnish).* Finnish Medical Journal 2018. **73**(7): p. 438-44.
12. Gissler, M. and J. Haukka, *Finnish health and social welfare registers in epidemiological research.* Norsk Epidemiologi, 2004. **14**(1): p. 113-120
13. Harkko, J., et al., *Socioeconomic Differences in Occupational Health Service Utilization and Sickness Absence Due to Mental Disorders: A Register-Based Retrospective Cohort Study.* Int J Environ Res Public Health, 2020. **17**(6).
14. Martimo, K.-P. and J. Mäkitalo, *The status of occupational health services in Finland and the role of the Finnish Institute of Occupational Health in the development of occupational health services. Report for the international evaluation of the Finnish Institute of Occupational Health (FIOH)*. 2014, Finnish Institute of Occupational Health: Helsinki.
15. Lopez Bernal, J., S. Cummins, and A. Gasparrini, *Interrupted time series regression for the evaluation of public health interventions: a tutorial.* Int J Epidemiol, 2017. **46**(1): p. 348-355.
16. Lopez Bernal, J., S. Cummins, and A. Gasparrini, *The use of controls in interrupted time series studies of public health interventions.* Int J Epidemiol, 2018. **47**(6): p. 2082-2093.
17. Degli Esposti, M., et al., *Can synthetic controls improve causal inference in interrupted time series evaluations of public health interventions?* Int J Epidemiol, 2020.
18. Hojsgaard, S., U. Halekoh, and J. Yan, *The R Package Geepack for Generalized Estimating Equations.* Journal of Statistical Software, 2006. **15**(2): p. 1-11.
19. Gelman, A. and J. Hill, *Data Analysis Using Regression and Multilevel/Hierarchical Models*. 2007: Cambridge University Press.

For peer review only

For peer review only - http://bmjopen.bmj.com/site/about/guidelines.xhtml

20. Xiao, H., O. Augusto, and B.H. Wagenaar, *Reflection on modern methods: a common error in the segmented regression parameterization of interrupted time-series analyses.* Int J Epidemiol, 2020.
21. Shumway, R.H. and D.S. Stoffer, *Time Series Analysis and Its Applications: With R Examples*. 4th ed. 2017: Springer.
22. *Official statistics of Finland: Index of wage and salary earnings 2018, 3rd quarter*. 2018; Available from: https://www.stat.fi/til/ati/2018/03/index_en.html.
23. Rossignol, M., et al., *Coordination of primary health care for back pain. A randomized controlled trial.* Spine (Phila Pa 1976), 2000. **25**(2): p. 251-8; discussion 258-9.
24. Becker, A., et al., *Effects of two guideline implementation strategies on patient outcomes in primary care: a cluster randomized controlled trial.* Spine (Phila Pa 1976), 2008. **33**(5): p. 473-80.
25. Bussieres, A.E., et al., *Impact of imaging guidelines on X-ray use among American provider network chiropractors: interrupted time series analysis.* Spine J, 2014. **14**(8): p. 1501-9.
26. Leece, P., et al., *Improving opioid guideline adherence: evaluation of a multifaceted, theory-informed pilot intervention for family physicians.* BMJ Open, 2020. **10**(1): p. e032167.
27. French, S.D., et al., *Evaluation of a theory-informed implementation intervention for the management of acute low back pain in general medical practice: the IMPLEMENT cluster randomised trial.* PLoS One, 2013. **8**(6): p. e65471.
28. Al Zoubi, F.M., et al., *The effectiveness of interventions designed to increase the uptake of clinical practice guidelines and best practices among musculoskeletal professionals: a systematic review.* BMC Health Serv Res, 2018. **18**(1): p. 435.
29. Zhelev, Z., et al., *Effectiveness of interventions to reduce ordering of thyroid function tests: a systematic review.* BMJ Open, 2016. **6**(6): p. e010065.
30. Timmermans, S. and A. Mauck, *The promises and pitfalls of evidence-based medicine.* Health Aff (Millwood), 2005. **24**(1): p. 18-28.
31. McCluskey, S., A.K. Burton, and C.J. Main, *The implementation of occupational health guidelines principles for reducing sickness absence due to musculoskeletal disorders.* Occup Med (Lond), 2006. **56**(4): p. 237-42.
32. Buyle, F., et al., *Implementation of guidelines for sequential therapy with fluoroquinolones in a Belgian hospital.* Pharm World Sci, 2010. **32**(3): p. 404-10.
33. Shadish, W.R., T.D. Cook, and D.T. Campbell, *Quasi-experimental experiments: Interrupted time-series designs*, in *Experimental and quasi-experimental designs for generalized causal inference*. 2002, Houghton Mifflin Company: Boston, New York. p. 623.

For peer review only

For peer review only - http://bmjopen.bmj.com/site/about/guidelines.xhtml

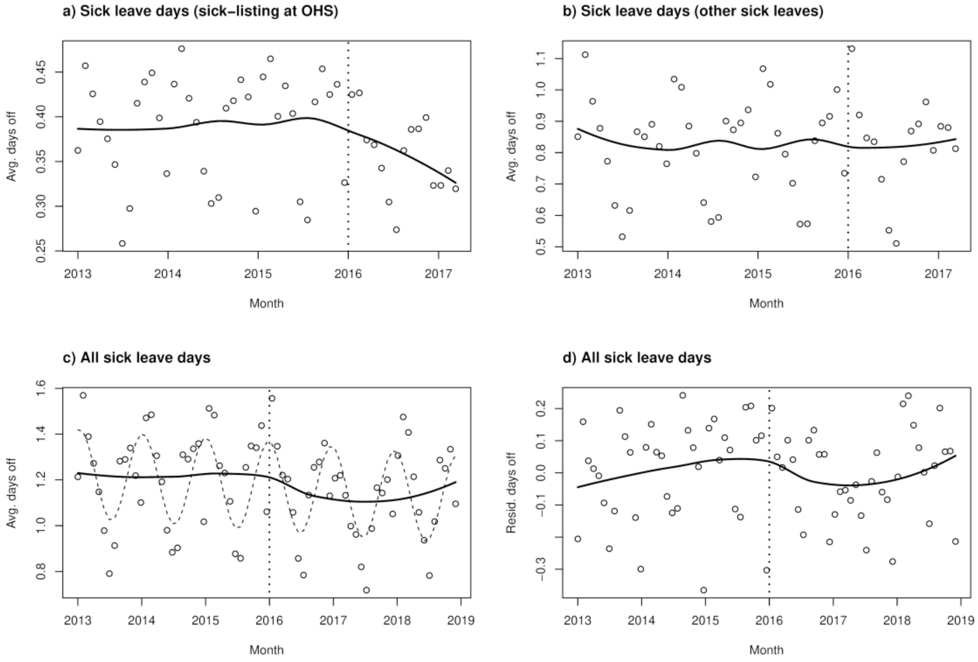

Figure 1: Model fits. Monthly averages of the number of all sick leave days. Intervention status could be specified until 21st April 2017 (Figures a) and b)). In Figures c) and d) the intervention status is not differentiated. Vertical dashed lines indicate the time point of intervention (all figures). Circles denote to observed averages, thick lines to their local regression fits (smoothed averages) (all figures), and thin dashed line to annual periodic variation (Figure c)).

173x120mm (800 x 800 DPI)

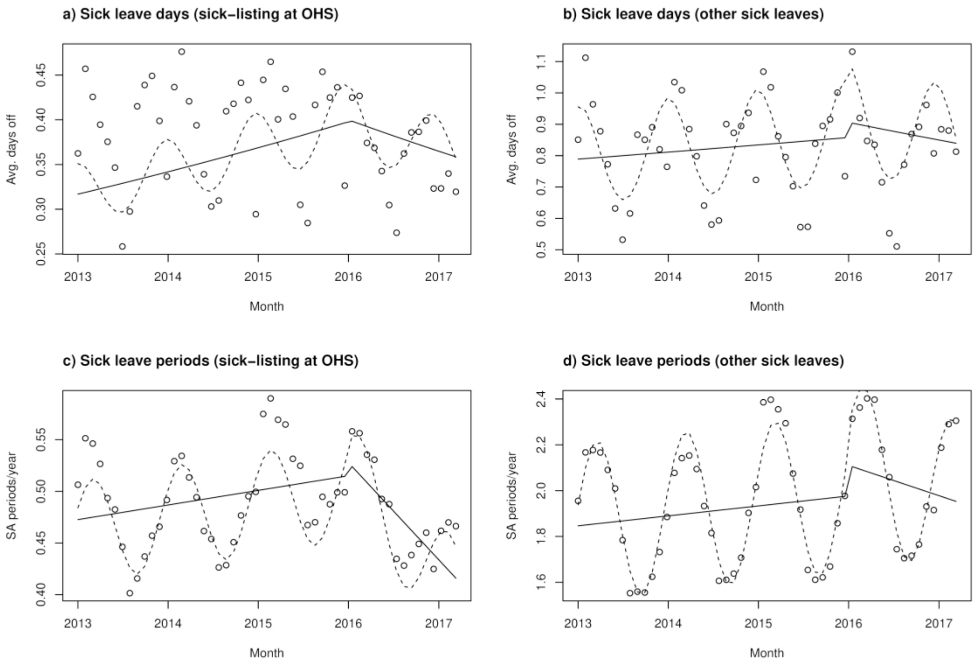

Figure 2: Model fits. Monthly averages of the number of all sick leave days (figures a) and b)) and periods (figures c) and d)). Observed data points denote to simple monthly averages and the lines represent GEE-model fits to employee-level data. The dashed line represents the full model prediction, including annual variations, whereas the solid line is the ITS-part of the model.
ITS=Interrupted time series analysis; GEE= generalised estimation equation

173x120mm (800 x 800 DPI)

Low back (lumbar back) pain, lumbago, M54.5

Lumbago is a very common ailment that everyone experiences at some point in their lives. It involves pain, aching and stiffness in the lumbar back. **It is almost always a harmless and benign condition**, which gets better by itself, but often recurs in following months. However, even if it recurs, its prognosis is good. The pain may begin suddenly in a harmless situation but may also develop gradually.

- **As a rule, lumbago gets better in a few days**. For more than 90% of sufferers, back pain is completely gone within 4–6 weeks.
- Signs of serious back disorders (continuous and progressively increasing backache even at rest, fever, weight loss, high-energy trauma, osteoporosis, changes in urinary and bowel continence, cancer history) can be ruled out using simple methods.

Assessing the need for sick leave:

○ According to new recommendations, sick leave may not at all necessary. There is no medical evidence that physically strenuous work slows down the healing of ordinary back pain or that it is associated with a risk of the condition continuing or of further injury.

○ **If sick leave is deemed necessary**: The patient's ability to do physically light work (only some lifting, bending and turning movements) may be **reduced for a few days or a week at the most.**

○ The patient's ability to perform physically strenuous work (which includes lifting, bending and turning movements) may be reduced for **two weeks at the most** (+ 2 weeks of partial sick leave if necessary), mainly because of difficulty lifting heavy objects. **Only after this does the checklist below apply.**

- **Temporary reduction and modification of pain-provoking factors are recommended.**
- Despite low back pain, working is not dangerous, and as a rule, continuing to work and not avoiding physical strain speeds up healing.
  - **Patients should be advised to use their backs normally – this is not dangerous**. Pain may become prolonged if the patient remains passive and afraid to move. Physical activity is beneficial; excessive resting is harmful. Moderate pain during exercise is not dangerous.
  - It is advisable to initially treat pain with sufficiently high doses of paracetamol and/or anti-inflammatory drugs. Strong painkillers (opiates) or muscle relaxants have not shown to be beneficial.
  - If the pain becomes prolonged, manipulation, massage or acupuncture may be beneficial.
  - In cases of prolonged pain, diverse drug-free pain management and pain threshold medicine can reduce pain sensitivity and improve pain tolerance.
  - Sleep and mood problems related to pain should be treated.

For peer review only - http://bmjopen.bmj.com/site/about/guidelines.xhtml

Checklist for when perceived work disability becomes prolonged:

DIAGNOSIS:

- Have **inflammatory diseases** (e.g., ankylosing spondylitis: Marie-Strümpell disease) been ruled out?
- Could the reason for the pain be a significant back **injury or previous cancer**?
- Does the pain or numbness radiate below the knee (**sciatic nerve entrapment)**?

WORK:

- Has work been modified to **temporarily reduce repeated lifting, leaning forward and twisted back postures, and work tasks that cause vibration**?
- Have p**sychosocial work load factors** (poor workplace atmosphere, lack of support from supervisor, excessive time pressure, overly demanding work, etc.) been identified? If so, has any attempt been made to change them?
- Has the employee **spoken to their supervisor** about their work and work ability?
- Have **occupational health services** been in contact with the supervisor **about work arrangements**?
- Has an **occupational physiotherapist** visited the workplace to advise on how to modify work tasks?

INDIVIDUAL FACTORS:

- Does the patient have any loading factors **outside of work** that may affect pain tolerance and coping with pain (crisis, grief, prolonged stress)?
- Does the patient do any **activities or** have any **hobbies outside of work** that may maintain the pain (sports, home renovations, long-distance driving, frequent use of laptop or mobile phone, gardening)?

TREATMENT:

- Have the **treatments** that the patient feels have best **benefit** been determined? Which treatments **make the pain worse**?
- Has the patient received **self-care instructions**?
- Does the patient use **active means** to control their pain? Exercise?

For peer review only - http://bmjopen.bmj.com/site/about/guidelines.xhtml

These issues, ‘yellow flags’, increase the risk of prolonged pain and work disability in the case of back pain:

- Sleep problems
- Anxiety, depression
- Fear of permanent damage or worsening of pain
- Pain all over the body
- Passivity, avoidance behaviour
- Problems at work, poor job satisfaction

For peer review only

For peer review only - http://bmjopen.bmj.com/site/about/guidelines.xhtml

Reporting checklist for cohort study.

Based on the STROBE cohort guidelines.

Instructions to authors

Complete this checklist by entering the page numbers from your manuscript where readers will find each of the items listed below.

Your article may not currently address all the items on the checklist. Please modify your text to include the missing information. If you are certain that an item does not apply, please write "n/a" and provide a short explanation.

Upload your completed checklist as an extra file when you submit to a journal.

In your methods section, say that you used the STROBE cohortreporting guidelines, and cite them as:

von Elm E, Altman DG, Egger M, Pocock SJ, Gotzsche PC, Vandenbroucke JP. The Strengthening the Reporting of Observational Studies in Epidemiology (STROBE) Statement: guidelines for reporting observational studies.

For peer review only

		Reporting Item	Page Number
**Title and abstract**			
Title	#1a	Indicate the study's design with a commonly used term in the title or the abstract	1

Abstract	#1b	Provide in the abstract an informative and balanced summary of what was done and what was found	2
**Introduction**			
Background / rationale	#2	Explain the scientific background and rationale for the investigation being reported	4
Objectives	#3	State specific objectives, including any prespecified hypotheses	5
**Methods**			
Study design	#4	Present key elements of study design early in the paper	5
Setting	#5	Describe the setting, locations, and relevant dates, including periods of recruitment, exposure, follow-up, and data collection	6
Eligibility criteria	#6a	Give the eligibility criteria, and the sources and methods of selection of participants. Describe methods of follow-up.	5
Eligibility criteria	#6b	For matched studies, give matching criteria and number of exposed and unexposed	n/a
Variables	#7	Clearly define all outcomes, exposures, predictors, potential confounders, and effect modifiers. Give diagnostic criteria, if applicable	9
Data sources / measurement	#8	For each variable of interest give sources of data and details of methods of assessment (measurement). Describe comparability of assessment methods if there is more than	5, 6, 9

For peer review only

		one group. Give information separately for for exposed and unexposed groups if applicable.	
Bias	#9	Describe any efforts to address potential sources of bias	10
Study size	#10	Explain how the study size was arrived at	5
Quantitative variables	#11	Explain how quantitative variables were handled in the analyses. If applicable, describe which groupings were chosen, and why	9
Statistical methods	#12a	Describe all statistical methods, including those used to control for confounding	10
Statistical methods	#12b	Describe any methods used to examine subgroups and interactions	10
Statistical methods	#12c	Explain how missing data were addressed	n/a
Statistical methods	#12d	If applicable, explain how loss to follow-up was addressed	n/a
Statistical methods	#12e	Describe any sensitivity analyses	12
**Results**			
Participants	#13a	Report numbers of individuals at each stage of study—eg numbers potentially eligible, examined for eligibility, confirmed eligible, included in the study, completing follow-	12

For peer review only

		up, and analysed. Give information separately for for exposed and unexposed groups if applicable.	
Participants	#13b	Give reasons for non-participation at each stage	n/a
Participants	#13c	Consider use of a flow diagram	n/a
Descriptive data	#14a	Give characteristics of study participants (eg demographic, clinical, social) and information on exposures and potential confounders. Give information separately for exposed and unexposed groups if applicable.	21
Descriptive data	#14b	Indicate number of participants with missing data for each variable of interest	n/a
Descriptive data	#14c	Summarise follow-up time (eg, average and total amount)	12
Outcome data	#15	Report numbers of outcome events or summary measures over time. Give information separately for exposed and unexposed groups if applicable.	22
Main results	#16a	Give unadjusted estimates and, if applicable, confounder-adjusted estimates and their precision (eg, 95% confidence interval). Make clear which confounders were adjusted for and why they were included	23
Main results	#16b	Report category boundaries when continuous variables were categorized	n/a
Main results	#16c	If relevant, consider translating estimates of relative risk into absolute risk for a meaningful time period	n/a

For peer review only

Other analyses	#17	Report other analyses done—e.g., analyses of subgroups and interactions, and sensitivity analyses	12, 23
**Discussion**			
Key results	#18	Summarise key results with reference to study objectives	16
Limitations	#19	Discuss limitations of the study, taking into account sources of potential bias or imprecision. Discuss both direction and magnitude of any potential bias.	18
Interpretation	#20	Give a cautious overall interpretation considering objectives, limitations, multiplicity of analyses, results from similar studies, and other relevant evidence.	17
Generalisability	#21	Discuss the generalisability (external validity) of the study results	17
**Other Information**			
Funding	#22	Give the source of funding and the role of the funders for the present study and, if applicable, for the original study on which the present article is based	16–19

The STROBE checklist is distributed under the terms of the Creative Commons Attribution License CC-BY. This checklist was completed on 12. November 2020 using https://www.goodreports.org/, a tool made by the EQUATOR Network in collaboration with Penelope.ai

For peer review only

BMJ Open

An intervention targeted at physicians' treatment of musculoskeletal disorders and sickness certification: An interrupted time series analysis

Journal:	*BMJ Open*
Manuscript ID	bmjopen-2020-047018.R2
Article Type:	Original research
Date Submitted by the Author:	23-Sep-2021
Complete List of Authors:	Kausto, Johanna; Finnish Institute of Occupational Health<br>Rosenström, Tom Henrik; University of Helsinki Faculty of Medicine, Department of Psychology and Logopedics<br>Ervasti, Jenni; Finnish Institute of Occupational Health<br>Pietiläinen, Olli; University of Helsinki, Department of Public Health<br>Kaila-Kangas, Leena; Finnish Institute of Occupational Health<br>Rahkonen, Ossi; University of Helsinki, Department of Public Health<br>Harkko, Jaakko; University of Helsinki Faculty of Social Sciences<br>Vaananen, Ari; Finnish Institute of Occupational Health<br>Kouvonen, Anne; University of Helsinki, Faculty of Social Sciences; Queen's University Belfast, Administrative Data Research Centre<br>Lallukka, Tea; University of Helsinki, Department of Public Health
**Primary Subject Heading**:	Public health
Secondary Subject Heading:	Evidence based practice, Occupational and environmental medicine
Keywords:	OCCUPATIONAL & INDUSTRIAL MEDICINE, PUBLIC HEALTH, Musculoskeletal disorders < ORTHOPAEDIC & TRAUMA SURGERY

SCHOLARONE™
Manuscripts

I, the Submitting Author has the right to grant and does grant on behalf of all authors of the Work (as defined in the below author licence), an exclusive licence and/or a non-exclusive licence for contributions from authors who are: i) UK Crown employees; ii) where BMJ has agreed a CC-BY licence shall apply, and/or iii) in accordance with the terms applicable for US Federal Government officers or employees acting as part of their official duties; on a worldwide, perpetual, irrevocable, royalty-free basis to BMJ Publishing Group Ltd ("BMJ") its licensees and where the relevant Journal is co-owned by BMJ to the co-owners of the Journal, to publish the Work in this journal and any other BMJ products and to exploit all rights, as set out in our licence.

The Submitting Author accepts and understands that any supply made under these terms is made by BMJ to the Submitting Author unless you are acting as an employee on behalf of your employer or a postgraduate student of an affiliated institution which is paying any applicable article publishing charge ("APC") for Open Access articles. Where the Submitting Author wishes to make the Work available on an Open Access basis (and intends to pay the relevant APC), the terms of reuse of such Open Access shall be governed by a Creative Commons licence – details of these licences and which Creative Commons licence will apply to this Work are set out in our licence referred to above.

Other than as permitted in any relevant BMJ Author's Self Archiving Policies, I confirm this Work has not been accepted for publication elsewhere, is not being considered for publication elsewhere and does not duplicate material already published. I confirm all authors consent to publication of this Work and authorise the granting of this licence.

For peer review only

BMJ OPEN
Public health research

An intervention targeted at physicians' treatment of musculoskeletal disorders and sickness certification: An interrupted time series analysis

Johanna Kausto[1], Tom Rosenström[1, 2], Jenni Ervasti[1], Olli Pietiläinen[3], Leena Kaila-Kangas[1], Ossi Rahkonen[3], Jaakko Harkko[4], Ari Väänänen[1], Anne Kouvonen[4,5], Tea Lallukka[3]

1 Finnish Institute of Occupational Health, Helsinki, Finland

2 Department of Psychology and Logopedics, Faculty of Medicine, University of Helsinki, Helsinki, Finland

3 Department of Public Health, University of Helsinki, Helsinki, Finland

4 Faculty of Social Sciences, University of Helsinki, Helsinki, Finland

5 Administrative Data Research Centre - Northern Ireland, Queen's University Belfast, Belfast, UK

*Corresponding author:

Johanna Kausto

Specialized Researcher

Finnish Institute of Occupational Health
PL 18
00032 TYÖTERVEYSLAITOS

FINLAND

johanna.kausto@ttl.fi

tel. + 358 30 474 2509, + 358 43 8200 698

Counts: Abstract (259), main text (4623), Tables (3), Figures (2)

Keywords: Sick leave [MeSH], Practice guidelines [MeSH], Occupational Health Service [MeSH], Musculoskeletal Diseases [MeSH]

For peer review only

Abstract

Objectives: An intervention was carried out at the occupational health care services (OHS) of the City of Helsinki beginning in 2016. We investigated the association between the intervention and employee sick leaves applying interrupted time-series analysis.

Design: Register-based cohort study with a quasi-experimental study design.

Setting: Employees of the City of Helsinki.

Participants: We analysed individual-level register-based data on all employees who were employed by the City for any length of time between 2013 and 2018 (a total 86,970 employees and 3,014,075 sick leave days). Sick leave days and periods that were OHS-based constituted the intervention time series and the rest of the sick leave days and periods contributed to the comparison time series.

Intervention: Provided recommendations for physicians on managing pain and prescribing sick leave for low back, shoulder, and elbow pain

Outcome measures: Number of sick leave days per month and sick leave periods per year.

Results: For all sick leave days prescribed in OHS, there was no immediate change in sick leave days, whereas a gradual change decreasing the number of OHS-based sick leave days was detected. On average, the intervention was estimated to have saved 2.5 sick leave days per year per employee. For other sick leave days, there was an immediate increase in the level of sick leave days after the intervention and a subsequent gradual trend decreasing the number of sick leave days.

Conclusions: The intervention may have reduced employee sick leaves and therefore it is possible that it had led to direct cost savings. However, further evidence for causal inferences is needed.

For peer review only

Article Summary

Strengths and limitations of this study

- This study presents novel information on the association between introducing recommendations for management of pain and prescribing sick leave for musculoskeletal disorders, on employee sick leave.

- Comprehensive individual level data from employer registers were linked to individual level data from national registers.

- We applied interrupted time series analysis with a comparison time series which is a strong quasi-experimental design, recommended for examining population-level interventions.

-Intervention status was not obtainable for the entire follow-up time, and information on diagnoses was available only for sick leave periods that lasted over 11 calendar days. As all employees could be prescribed sick leave both at OHS and elsewhere, the validity of the comparison group could be debated. However, only the professionals in the intervention group were exposed to the intervention and the time series for the intervention and comparison groups were analysed separately.

For peer review only - http://bmjopen.bmj.com/site/about/guidelines.xhtml

Introduction

Musculoskeletal disorders (MSD) are highly prevalent among working age population, and they result in extensive disability costs both for the individuals and the society. In Finland in 2019, 27% of the days of sick leave periods lasting at least 11 calendar days were due to MSD [1] . Thus, sick leave is typically granted for musculoskeletal pain symptoms, such a s back, shoulder or elbow pain. However, scientific evidence does not support the beneficiality of long sick leaves. For example, it has been shown that when having low back pain, from a point of view of recovery, staying active is better than bed rest [2, 3].

Primary care doctors' advice can have a major impact on whether an individual takes sick leave and for how long [4]. A follow-up study found that visiting a medical specialist due to a musculoskeletal disorder was associated with a delayed full return to work irrespective of the severity of ailment [5]. Furthermore, earlier studies have found that sick listing (sick leave prescribing) practices vary a lot across physicians [6, 7]. A Finnish study showed that the differences in the length of prescribed sick leaves can be up to 8-fold [8]. In line with this, a survey found that Finnish physicians experienced that they needed more information, guidance and training on sick listing [9].

Against this background, an intervention was carried out at Occupational Health Helsinki, which provides occupational health care services (OHS) for the City of Helsinki employees. The intervention was launched in 2016. Introducing and increasing the use of shared guidelines on the management of pain and work disability and prescribing sick leave for low

For peer review only - http://bmjopen.bmj.com/site/about/guidelines.xhtml

back, shoulder and elbow pain was the central element in the intervention. To investigate the association of this intervention with employee sick leaves, we carried out prospective controlled time series and binomial regression analyses using register-based data.

Methods

Study design and population

Data were collected as a part of the Helsinki Health Study [10]. The study population includes a sample of the employees of the City of Helsinki. Women represent 70% of the employees who work in general local administration, social and health care, education and culture, public transport, and technical services. All employees share the same personnel administration, policies, and occupational health care services [10]. Employees can use the services (including primary care services for other than work-related conditions) of their OHS provider, Occupational Health Helsinki; or they can seek treatment from public or private health care. The employees of the City of Helsinki can use self-certification for sick leaves lasting up to five days. In the case of self-certification, a medical certificate is not needed, but the employee notifies the supervisor when taken ill. In 2016, self-certification was mostly used among young employees (under 30 years of age) [11].

For the purposes of this study, individual-level register-based data of all the employees who were employed by the City for any length of time between 2013 and 2018 (n= 86,970) were analysed. Background information on participants were drawn from employer's registers. Information on all sick leave periods (start and end dates and diagnostic codes (ICD-10)) during this time were derived from employer's registers and the national sickness insurance

For peer review only - http://bmjopen.bmj.com/site/about/guidelines.xhtml

For peer review only

register of the Social Insurance Institution of Finland (SII). ICD-10 codes for sick leave periods were available from the SII for sick leave periods lasting more than 11 calendar days. Data on chronic somatic and psychiatric illnesses and the purchases of prescribed psychotropic medication were obtained from the Register of Special Reimbursement for Medication Purchases (SII). Data from these three registers were linked based on unique individual numbers of the participants. Such numbers are assigned to each citizen of Finland at birth and for migrants when they get a residence permit, and they are common to all administrative registers, enabling extensive record linkages [12]. The study follows the Helsinki Health Study (HHS) protocol in line with the University of Helsinki's guidelines and the EU and Finnish data legislation. The ethics committees of the Department of Public Health, the University of Helsinki and the health authorities of the City of Helsinki have approved the HHS study. The City of Helsinki and register holders have given permissions for data linkage.

Patient and Public Involvement

As this is a register study, no patients were directly involved.

Study context

Occupational Health Care in Finland

In the Finnish occupational health care service (OHS) system all employees are entitled to statutory preventive OHS provided by the employer. Most employers offer state-subsidised free primary care services for their employees on top of the statutory preventive OHS. OHS providers in Finland provide primary care services to employees in a way that is comparable

For peer review only - http://bmjopen.bmj.com/site/about/guidelines.xhtml

to primary care in international comparisons in substance, but differs in that the service is targeted only to the working population [13]. The statutory tasks of OHS include assessment of the health and safety aspects of the work, assessment and monitoring of employees' health and work ability, making initiatives for improvement and monitoring their implementation, advice and guidance, monitoring employees with disabilities and referring them to rehabilitation, co-operation with representatives of other health care services and social insurance, participation in organising first aid at the workplace, participation in activities that maintain work ability, and monitoring the quality and impact of occupational health care activities [14].

Intervention

Occupational Health Helsinki provides occupational health care to the almost 40,000 employees working annually in the City departments. The employees represent hundreds of different occupations, and a rather large supply of health care services supporting the employees' work ability and the functioning of work units is offered. The Occupational Health Helsinki staff consists of 150 health care professionals.

The aims of the intervention were to increase the quality and effects of occupational health care, to increase the use of mutually created and shared guidelines based on available scientific evidence in assessing work disability, to provide a practical instrument for physicians in managing pain and prescribing sick leave in the form of recommendations, to help physicians to take into account the complexity of the association between pain and work disability, and to provide up-to-date information to the patient about their pain

For peer review only - http://bmjopen.bmj.com/site/about/guidelines.xhtml

condition, its treatment and how it may affect work and work ability. The goal was to reduce employee sick leaves and improve work ability.

Recommendations on work disability management and prescribing sick leave for low back pain, shoulder pain and elbow pain were launched first in January 2016 and they were the central and the most noticeable component in the intervention. Recommendations were launched in a form of three guideline papers (one for each diagnostic category). Training regarding the implementation of the guidelines was organised in OHS for physicians and all other relevant health care professionals such as physiotherapists and nurses. Short educational sessions were offered, and an e-learning course and some coaching sessions were led by a pain specialist. An intensive follow-up of the sick leave trends was started in OHS. For instance, recent trends of sick leave were reported in the monthly meetings for the physicians. All new relevant OHS employees were briefed about the guidelines.

As an example, in the case of low back pain, the recommendation (Appendix 1) emphasised that the condition is very common and nearly all people experience a disabling low back pain (i.e. pain, ache and stiffness in the area of lower back) at some point in their lives. The condition is usually benign in nature, heals spontaneously in a few days, and 90% of the patients will fully recover in 4 to 6 weeks. Still, possible serious causes for back pain should be ruled out. In sick listing the following points should be considered: In many cases sick leave is not needed; There is no indication that physically strenuous work would slow down recovery; If sick listing is necessary, a few days up to a week should be sufficient in light duties; If work includes heavy lifting, bending or rotation, two weeks of full time sick leave may be needed; Factors that provoke pain should be temporarily modified; Factors that suggest an increased risk for prolonged back pain and work disability include sleeping

For peer review only - http://bmjopen.bmj.com/site/about/guidelines.xhtml

problems, depression and anxiety, fear avoidance beliefs, multisite pain, and problems at the workplace and dissatisfaction with work. The recommendations included a checklist for the physicians to be used at the appointment.

Intervention status

Sick leave periods that were OHS-based (if a sick leave was preceded by a likely related appointment at OHS within 11 days, it was approximated that sick-listing had taken place at OHS) constituted the intervention time series and the rest of the sick leave periods (no appointment at OHS, but appointment and sick-listing had taken place elsewhere or the employee had used self-certification, in which case there had been no medical appointment at all) contributed to the comparison time series. The available registers contained information of appointments in the OHS until 20th April 2017.

Outcome

We calculated the number of sick leave days per month and sick leave periods per year.

Covariates

Covariates that were available in the registers and were regarded as potential confounders included age, sex, occupational class (based on occupational title and categorised as upper grade non-manual employees, intermediate grade non-manual employees, lower grade non-manual employees and manual workers), job contract (permanent, temporary) valid 1st January 2016, chronic somatic illnesses (derived from the Register of Special

For peer review only - http://bmjopen.bmj.com/site/about/guidelines.xhtml

Reimbursement for Medication Purchases, and included diabetes, heart disease, rheumatoid arthritis, chronic asthma, stage 2 hypertension, Parkinson's disease, epilepsy, uremia, bowel disease, multiple sclerosis disease, and diseases of pancreas and categorized as 0=no, 1=yes) valid 1st January 2016, and purchases of prescribed psychotropic medication (Anatomic Therapeutic Chemical, ATC, classification N06 or N05 categorised as 0=no, 1=yes) between 1st November 2015 and 1st November 2016.

Statistical analyses

We used an interrupted time series (ITS) design with a comparison time series [15-17]. The observational unit in the analyses was a sick leave day or period. The ITS analysis uses time-series (a continuous sequence of observations on a population-level taken repeatedly at equal intervals over time) for a specific outcome to establish an underlying trend which is "interrupted" by an intervention at a specific point of time. A strength of ITS analyses is that they are generally not affected by typical confounding factors (such as age distribution or socioeconomic status) as they model a time trend in a context where the population composition (in terms of the confounding factors) remains rather constant before vs. after the intervention. Measured time varying confounders can be controlled for in the regression models. Unmeasured or unknown time varying confounders can be controlled by adding a comparison group (not exposed to the intervention) [15]. It has been recommended that the covariate balance between the intervention and comparison time series is explored, though it is not a prerequisite for the analysis [16].

Our integer-valued (count, or proportion) outcome variable was days of sick leave per month (range 0–30, or from 0 to days exposed within the 30-day period, if not with the

For peer review only - http://bmjopen.bmj.com/site/about/guidelines.xhtml

employer for the full 30-day period), recorded separately for each month. Although each employee was followed up for multiple months, different employees may have different time average rate of sick leave days, introducing statistical dependencies to the observations. As our interests pertained to a population-level associations , within employee clustering in the outcome was considered a nuisance variable and its associations were modeled using generalised estimating equations [18]. Given the data characteristics above, the outcome was modeled using generalised (binomial) linear regression model [19] and the models were estimated with generalised estimating equations. Although our data was in the form of monthly counts, an exponentiated binomial regression coefficient can be interpreted as an odds ratio (OR) for a sick-leave day (vs. no sick leave) for a randomly chosen day, adjusting for the other covariates in the model. As a sensitivity analysis, we also tested quasi-binomial generalised linear model with usual maximum likelihood estimation, which gave similar results with narrower confidence intervals.

The main test of our hypothesis pertained to the model equation

$$g^{-1}(Y) = \beta_0 + \beta_{slope}(m - m_0) + \beta_{immediate}X + \beta_{gradual}X(m - m_0),$$

where Y is the outcome, g the logit-link function, X is 1 for all months after the intervention launch date (1st of January 2016) and 0 before it, and $(m - m_0)$ is time (months, centered around the time of intervention [20] , m_0). Here, β_{slope} is the linear trend estimate before the intervention, whereas $\beta_{slope} + \beta_{gradual}$ is the trend after the intervention took place. The intercept before the intervention is β_0 and that after the intervention $\beta_0 + \beta_{immediate}$. Thus, $\beta_{immediate}$ is the immediate trend after the intervention and $\beta_{gradual}$ the gradual trend after the intervention that accumulates at the rate $\beta_{gradual}$ per month after the intervention (for the remaining observation period). Besides this usual ITS setting, we took into account

For peer review only - http://bmjopen.bmj.com/site/about/guidelines.xhtml

the annual fluctuation of sick leave by modelling an annual trend estimating coefficients for two additional covariates with values $\sin(m2\pi/12)$ and $\cos(m2\pi/12)$, where the factor $2\pi/12$ scales monthly data points m to the annual cycles [21] .

Because our outcome data timepoints (months) could include sick leave days from prescribed at OHS and other sources, defining a covariate for intervention group membership would have required us to prioritise either OHS or other sources. It is typical in CITS design to include a binary covariate that gets a value 1 when a unit belongs to the intervention group and value 0 when in the control group. In this study, participants had sick leaves both from OHS and non-OHS sources. Instead of formal testing of such covariate, we conducted a separate ITS-design and analyses for the intervention and comparison groups. Both crude models and models adjusted for the covariates were fitted. Analyses were run separately for men and women, for short (<= 11 calendar days) and longer (> 11 calendar days) sick leave periods, and for sick leave periods (>=11 calendar days) in the diagnostic categories of back pain (ICD-10 M54.5), shoulder pain (ICD-10 M75) and elbow pain (ICD-10 M77.1).

As the sick leave recommendations potentially were associated with both the number of sick leave days and periods, the number of consecutive sick leave periods were computed within index month plus 5 immediately preceding months. Monthly sample averages of numbers of periods were investigated with otherwise similar linear models as in above but using a six-month moving-average error structure. The “arima” function of R version 3.5.1 (2018-07-02) was used. Local regression lines for figures were drawn using loess-function of “stats” R package, version 3.5.1, with the default options. The same software was used for all the analyses.

For peer review only - http://bmjopen.bmj.com/site/about/guidelines.xhtml

Results

Descriptive results

Our data included information on sick leaves of a total of 86,970 employees covering more than 73 months. Due to workforce turnover, the monthly sample size of employees averaged at 41,289, with a minor variance across months (min 37,567, max 45,520; SD 2,054). Overall, 70% of all employees were on sick leave at least once during the follow-up and 33% of the employees were prescribed sick leave at OHS at least once between 1st January 2013 and 21st April 2017. Of those employees who were prescribed sick leave at OHS, 95% were prescribed sick leave also elsewhere (or they used self-certification). Of those who were prescribed sick leave elsewhere (or used self-certification) at least once, 46% were prescribed sick leave also at OHS. Of all employees, 30% were not on sick leave at all. On average, employees were prescribed 0.36 days of sick leave per month at OHS and 0.82 sick leave days per month were prescribed elsewhere or were self-certified. The respective mean numbers of sick leave periods per employee per year were 0.49 and 1.96.

Descriptive statistics for all employees, employees with sick leaves prescribed at OHS and employees with other sick leaves (prescribed elsewhere or self-certificated) are shown in Table 1 and Table 2. Those employees who were prescribed sick leave at OHS at least once by 21st April 2017, were more often permanently employed, older, female, and intermediate or lower level non-manual workers (Table 1).

Monthly averages of the number of all sick leave days

For peer review only - http://bmjopen.bmj.com/site/about/guidelines.xhtml

Since it is generally recommended to draw simple plots of ITS analyses before entering into more complex model fitting [15, 16], we started by examining the monthly average days of sick leave per employee. Based on a visual inspection of monthly averages, the number of all sick leave days prescribed at OHS declined shortly after the intervention had started in 2016 (Figure 1a). and similar temporal changes were not observed for other sick leave periods (Figure 1b). After 21st April 2017, our register data could not differentiate between sick leave days that were OHS-based and other sick leave days, but we had a longer stretch of data when counting in all sick leaves. A decline in sick leave days after intervention was seen in a visual inspection of all sick leaves (thick line for local-regression smoother), as well as annual periodic variation (thin dashed line in Figure 1c). When removing cyclic annual variation and a linear trend, the visual suggestion of an association between the intervention and sick leaves remained. However, sick leaves appeared to return to preceding rates towards the end of the longest available follow-up data (in 2018) (Figure 1d). It should be noted that Panels in Figures 1 and 2 are not comparable to each other as Y-axis are different. The figures illustrate how the trend in sick leave days changes at the time of the intervention rather than compare absolute values. Due to relatively small change in trend, similar Y-axis would not illustrate the trend.

Formal modeling (adjusting for seasonal variation) did not reveal an immediate diminishing trend($\beta_{immediate}$ = 0.01, 95% CI = (-0.052–0.072)), but did detect a gradual diminishing trend ($\beta_{gradual}$ = -0.007, 95% CI = (-0.013– -0.001)) in the number of OHS-based sick leave days. The corresponding numbers were similar in direction but not statistically significant in the comparison group ($\beta_{immediate}$ = 0.068, 95% CI = (-0.065–0.201)), and ($\beta_{gradual}$ = -0.009, 95% CI = (-0.022– 0.004)) (data not shown). These formal findings would support a gradual

For peer review only - http://bmjopen.bmj.com/site/about/guidelines.xhtml

diminishing trend after the intervention, but we were able to achieve a greater statistical power by accessing the employee-level data instead of the above-discussed monthly averages.

Results on employee-level data: The number of all sick leave days

We modelled the number of sick leave days using binomial regression on monthly sick leave days per employee. Figures 2 a and b illustrate the model predictions for the number of all sick leave days. For convenient illustration, we plotted the model estimates overlaid on the above discussed monthly averages. These models use 2,160,445 observations from 75,962 individuals instead of 52 monthly averages. The exact binomial regression coefficients (with logit-link function) as well as odds ratios for a sick leave day at index month vs. preceding month are presented in Table 3.

We observed a clear gradual diminishing trend in the number of all-cause OHS-based sick leave days (first model, Table 3). A similar gradual trend was found in the comparison group (sick-listing elsewhere or self-certification), but that was offset by an increase in the level of the number of sick leave days at the time of intervention. Adjusting for the covariates (Table 3, model 2) did not affect the estimates significantly. We additionally ran the analyses separately for men and women (not shown in the table). The results were in line with the first model (total sample). The association between the intervention and sick leaves found in all-cause sick leave was not paralleled in diagnostic group-specific findings (models 4 to 6 in Table 3).

While a gradual diminishing trend of sick leave days of odds ratio 0.986 may appear small, we can illustrate the association between the intervention and sick leave days in a practical

For peer review only - http://bmjopen.bmj.com/site/about/guidelines.xhtml

way as follows. Assuming employees on average have 1.2 sick-leave days per month (cf. Fig. 1c), their baseline risk rate per day would be 0.04. Small risk ratios closely match odds ratios. Thus, if the other factors have already been adjusted for, the intercept for a logistic model would be $\beta_0 = \log(0.04)$. Then the gradual intervention estimate of $OR_{gradual} = 0.986$ would imply that odds ratio of a sick-leave day one year after the intervention has reduced to $\exp(\beta_0 + 12 \times \log(OR_{gradual})) \approx 0.034$ and the risk rate of a sick-leave day to approximately 0.033. Thus, the gradual intervention estimate on average amounts to $(0.04 - 0.033) \times 30 \times 12 = 2.52$ avoided sick-leave days per year per employee. Expressed in another way, the intervention may have saved 2,520 sick-leave days per year per 1,000 employees.

Results on the employee-level data: The number of all sick leave periods

As a sensitivity analysis, we investigated the number of all-cause sick leave periods as an outcome. A sick leave period is a consecutive spell of sick leave days. We report results in units of periods per year per employee, although we computed periods per index month plus five preceding months (Figure 2 c and d). We analysed the monthly averages with a moving-average regression model. For all sick leave periods prescribed in OHS, we did not detect any immediate change in trend ($\beta_{immediate}$ = 0.017, 95% CI = (-0.011– 0.046)), but a gradual diminishing trend in the number of sick leave periods was found ($\beta_{gradual}$ = -0.009, 95% CI = (-0.013– -0.005)). The findings on the gradual change of trend were similar in the comparison group ($\beta_{gradual}$ = -0.014, 95% CI = (-0.026– -0.003)), whereas the immediate change in trend indicated that an increase of the sick leave periods occurred in the comparison group at the time of intervention ($\beta_{immediate}$ = 0.141, 95% CI = (0.067– 0.214)).

For peer review only - http://bmjopen.bmj.com/site/about/guidelines.xhtml

Discussion

In this register-based study among municipal employees, we analysed sick leave trends before and after an intervention targeted at physicians' management of musculoskeletal pain-related work disability and prescribing sick leave. The intervention was carried out in OHS setting. We examined monthly averages of all-cause sick leave days and found no immediate change in trend among OHS-based sick leave days but did detect a gradual decreasing trend in the number of OHS-based sick leave days by 2.5 days per year per employee. The corresponding findings were similar in direction but not statistically significant for the comparison time series (sick-listing elsewhere or self-certification). In the employee-level data (with a larger statistical power), we found a clear gradual decreasing trend in the number of OHS-based all-cause sick leave days. Similar gradual decreasing trend was found for the comparison time series, but that was offset by an increase in the level of sick leave days and periods at the time of intervention. A hypothetical organisation with 40,000 employees and an average direct cost 250 of EUR per sick-leave day (numbers similar to those of the City of Helsinki) [22], is likely to have resulted in substantial annual savings at the time of the intervention.

The knowledge base on physicians' sick-listing practices is accumulating. The present study suggests that there was an association between the intervention and the sick-listing practices of physicians working in Occupational Health Helsinki. There was a downward trend in the number of OHS-based sick leave days and periods. Nevertheless, the findings may also suggest that soon after the intervention carried out in OHS, the employees

For peer review only - http://bmjopen.bmj.com/site/about/guidelines.xhtml

increasingly sought treatment elsewhere (or used self-certification increasingly) as a rise in the level of other sick leaves was detected. This trend leveled down, however.

As far as we know, there are no previous studies investigating the effects of introducing guidelines on prescribing sick leaves (as compared to the situation where no guidelines exist). A few studies compare different ways of implementation [23, 24]. There are many previous studies on interventions attempting to change physicians' behaviour in some other areas of clinical practice. Findings show that success in changing the behaviour and adherence to different clinical guidelines varies. Some studies report success in behaviour change [25, 26], whereas other studies report limited success, or found interventions ineffective [27-29]. Moreover, a change in physician's behaviour does not automatically reflect in patient outcomes [28]. Guidelines have been found to be a necessary but an insufficient step in changing clinical care [30]. The importance of proactive implementation of guidelines and efforts to include the new practice into existing organisational procedures, which both were attempted in this intervention, have been emphasised to achieve sustainable results [31, 32]. As the context of the intervention is important, the results of an intervention in one environment cannot directly be applied to another context.

A strength of this study is its design based on register data on all employees. We could link comprehensive data from employer registers to data from national registers using personal identification numbers resulting in no loss to follow-up. The use of administrative registers in research always entails limitations as they are not typically collected for research purposes. Nevertheless, in general, the quality of registers in Finland as in other Nordic

For peer review only - http://bmjopen.bmj.com/site/about/guidelines.xhtml

countries is good and administrative registers offer unique opportunities and are frequently used in research.

Applied ITS analysis with a comparison time series has been recommended for analysing population-level interventions [15, 33]. A limitation was that the intervention status was approximated (an OHS-based sick leave was preceded by a likely relevant appointment at OHS) and the information was not available for the entire follow up period. The validity of the comparison group can be debated [17] as all employees could be prescribed sick leave both at OHS and elsewhere (or use self-certification in short sick leaves). However, only the OHS professionals were exposed to the intervention and the time series for the intervention and comparison groups were analysed separately. Thus, our analysis should be thought of as being in between ITS and CITS in what comes to scientific value of evidence.

Because the intervention was targeted at managing pain and prescribing sick leave from the first day of work disability, a limitation was that we had access to diagnostic data only for sick leave periods that lasted over 11 calendar days. This, together with the low base rate for specific diagnoses, may explain the result that the association between the intervention and sick leaves found in all-cause sick leaves was not replicated in diagnosis-specific findings of sick leaves (lasting over 11 calendar days) due to low back, shoulder, and elbow pain. Musculoskeletal disorders constitute a major cause for sick leave. The fact that an association between the intervention and sick leaves was seen among all-cause sick leaves (regardless of the length of the sick leave period) may also indicate that recommendations given for these three specific disorders influenced how the physicians managed work

For peer review only - http://bmjopen.bmj.com/site/about/guidelines.xhtml

disability and prescribed sick leave altogether. Unfortunately, we did not have information on part time sick leaves and for that reason we could not take this into account in the analyses. In Finland, partial sickness benefit can be granted only after 11 days of full-time sickness absence. Partial sickness benefit days comprised only 7% of all compensated sickness benefit days in Finland in 2016.

The recommendations for physicians constituted chronologically the first and the main element in the intervention. Direct access to physiotherapists without a doctor's referral was introduced in the following year, and thereafter it was not possible to differentiate the association between these different elements and sick leaves.

To conclude, we observed a gradual declining trend in the number of sick leave days and periods prescribed in OHS after the intervention. This is consistent with the intervention being effective, but the causality of the relationship is unclear due to similar findings for the comparison group. The estimated reduction of 2.5 sick leave days per year per employee could leave to substantial savings for a large employer such as the City of Helsinki. This can be considered as an economically relevant intervention, but further evidence for its causal interpretation is needed.

For peer review only - http://bmjopen.bmj.com/site/about/guidelines.xhtml

Figure legends

Figure 1: Model fits. Monthly averages of the number of all sick leave days. Intervention status could be specified until 21st April 2017 (Figures a and b). In Figures c and d the intervention status is not differentiated. Vertical dashed lines indicate the time point of intervention (all figures). Circles denote to observed averages, thick lines to their local regression fits (smoothed averages) (all figures), and thin dashed line to annual periodic variation (Figure c). Please note the differing scales in the figures.

Figure 2: Model fits. Monthly averages of the number of all sick leave days (figures a and b) and periods (figures c and d). Observed data points denote to simple monthly averages and the lines represent GEE-model fits to employee-level data. The dashed line represents the full model prediction, including annual variations, whereas the solid line is the ITS-part of the model. Please note the differing scales in the figures.

ITS=Interrupted time series analysis; GEE= generalised estimation equation

For peer review only

For peer review only - http://bmjopen.bmj.com/site/about/guidelines.xhtml

Aknowledgements: We would like to thank MD, Chief Medical Officer Eevamaija Tuovinen, MD, PhD Helena Miranda, and CEO, PhD Tiina Pohjonen, Occupational Health Helsinki for providing information of the intervention and its context.

Conflicts of Interest: The authors have no conflicts of interest to declare. The funders had no role in the design of the study; in the collection, analyses, or interpretation of data; in the writing of the manuscript, or in the decision to publish the results.

Funding: This work was supported by the Social Insurance Institution of Finland (grant # 9/26/2019 JK, TR, JE, AV and LK-K), The Finnish Work Environment Fund (grant #190081 JK, TR, JE, AV and LK-K), the Academy of Finland (grants #1294514 and #319200 OR and TL) and the Economic and Social Research Council (ESRC) (grant # ES/S00744X/1 AK).

Open access funding provided by the Finnish Institute of Occupational Health.

Author Contributions: Conceptualisation and methodology: JK, TR, OP, AV, EJ, OR, JH, AK, and TL; writing–original draft preparation: JK and TR; writing–review and editing: JK, TR, EJ, OP, LKK, OR, JH, AV, AK, TL; project administration: JK.

All authors have read and agreed to the published version of the manuscript.

"We used the **STROBE cohort checklist** when writing our report “An intervention targeted at physicians’ treatment of musculoskeletal disorders and sickness certification: Aninterrupted time series analysis”.

Ethics approval and consent to participate: The ethics committees of the Department of Public Health, the University of Helsinki and the health authorities of the City of Helsinki approved the Helsinki Health Study protocol. The ethical approval applies to the current study. The participants gave written informed consent to participate in the study.

Provenance and peer review: Not commissioned; externally peer reviewed.

Data sharing statement: No additional data are available.

For peer review only - http://bmjopen.bmj.com/site/about/guidelines.xhtml

Table 1. Descriptive statistics for all employees, employees with sick leaves prescribed at OHS[1] and employees with sick leave prescribed elsewhere or self-certified (other sick leaves)*.

Characteristic	Sick-listing at OHS (n= 25200)	Other sick leaves (n=52174)	All (n=75962)
Age (Mean, SD)	44.67 (12.32)	42.16 (13.02)	40.27 (14.08)
Men (%)	20	23	27
Occupational class (%)			
Upper grade non-manual employees	21	24	27
Intermediate grade non-manual employees	27	25	22
Lower grade non-manual employees	37	36	34
Manual workers	16	15	17
Job contract (%)			
Permanent	82	67	54
Prescribed reimbursed purchases of medication for chronic diseases (%)			
Yes	21	17	16
Prescribed reimbursed psychotropic medication (%)			
Yes	12	9	8

* Note that many employees were prescribed sick leave both at OHS and elsewhere (or used self-certification). The group “All” contains also employees with no sick leaves.

OHS=Occupational health care services.

For peer review only - http://bmjopen.bmj.com/site/about/guidelines.xhtml

Table 2. Monthly average numbers of sick leave days per month per employee by intervention status (years 2013–2017).

	Sick-listing at OHS[1]			Other sick leaves		
	Average	Range	SD	Average	Range	SD
All sick leave days	0.380	0–30	2.59	0.840	0–30	3.40
All short sick leave periods[a]	0.117	0–25	0.79	0.392	0–24	1.24
Sick leave for low back pain[b]	0.005	0–30	0.36	0.007	0–30	0.39
Sick leave for shoulder pain[b]	0.020	0–30	0.71	0.014	0–30	0.59
Sick leave for elbow pain[b]	0.004	0–30	0.28	0.002	0–30	0.19

OHS=Occupational health care services

[a] l<= 11 calendar days, [b] > 11 calendar days

For peer review only

Table 3. Binomial regression models predicting the number of sick leave days.

	Sick listing at OHS				Other sick leaves			
	Coefficient β	Std. error	OR[1)]	95% CI	Coefficient β	Std. error	OR[1)]	95% CI
Sick leave days (all periods, all DC:s) (Crude model)[2)] (n=2 160 445 employee-months, n=75,962 employees)								
Immediate change in trend	0.012	0.035	1.012	0.95–1.08	0.060**	0.018	1.062	1.03–1.10
Gradual change in trend	-0.014***	0.003	0.986	0.98–0.99	-0.008***	0.002	0.992	0.99–1.00
Linear trend before intervention (Slope)	0.006***	0.001	1.006	1.00–1.01	0.002***	0.001	1.002	1.00–1.00
Sick leave days (all periods, all DC:s) (Fully adjusted model)[2)] (n=2 148 566 employee-months, n= 74,701 employees)								
Immediate change in trend	-0.003	0.032	0.997	0.94–1.06	0.057**	0.018	1.059	1.02–1.10
Gradual change in trend	-0.010**	0.003	0.990	0.98–1.00	-0.007***	0.002	0.993	0.99–0.99
Linear trend before intervention (Slope)	0.007***	0.001	1.007	1.01–1.01	0.003***	0.001	1.003	1.00–1.01
Sick leave days (short periods, < 11 calendar days, all dg:s) [2)] (n=2 160 445 employee-months, n=75,962 employees)								
Immediate change in trend	0.105***	0.024	1.111	1.06–1.16	0.196***	0.010	1.217	1.19–1.24
Gradual change in trend	-0.020***	0.002	0.980	0.98–0.98	-0.009***	0.001	0.991	0.99–0.99
Linear trend before intervention (Slope)	0.001	0.001	1.001	1.00–1.00	-0.003***	<0.001	0.997	1.00–1.00
Sick leave days in low back pain (periods >= 11 calendar days) [3)] (n=2 160 445 employee-months, n=75,962 employees)								
Immediate change in trend	-0.547	0.340	0.579	0.30–1.13	-0.205	0.283	0.815	0.47–1.42
Gradual change in trend	0.040	0.035	1.041	0.97–1.12	-0.019	0.031	0.981	0.92–1.04
Linear trend before intervention (Slope)	-0.004	0.009	0.996	0.98–1.01	-0.004	0.007	0.996	0.98–1.01

OHS=Occupational health care services, DC=diagnostic code. OR=Odds ratio, CI=Confidence interval. *p < 0.05, **p < 0.01, *** p< 0.001.

[1)] Immediate intervention estimatet: Odds ratio was estimated for any employee to have any sick leave day after vs. before the intervention. Gradual interventionestimste: Odds ratio was estimated for any employee to have a sick leave day at index month vs. preceding month during the period after the intervention.[2)] Follow-up from 1st January 2013 to 21 st April 2017 [3)] Follow-up from 1st January 2013 to 31st December 2019. Adjusted for age, sex, occupational class, job contract, prescribed purchases of medication for chronic diseases, prescribed psychotropic medication.

For peer review only - http://bmjopen.bmj.com/site/about/guidelines.xhtml

Table 3. Binomial regression models predicting the number of sick leave days (Continued).

	Sick-listing at OHS				Other sick leaves			
	Coefficient β	Std. error	OR[1)]	95% CI	Coefficient β	Std. error	OR[1)]	95% CI
Sick leave days for shoulder pain (periods >= 11 calendar days)[3)] (n=2 160 445 employee-months, n=75,962 employees)								
Immediate change in trend	0.365	0.207	1.441	0.96–2.16	-0.426	0.292	0.653	0.34–1.16
Gradual change in trend	-0.023	0.023	0.977	0.93–1.02	0.013	0.025	1.013	0.97–1.06
Linear trend before intervention (Slope)	-0.004	0.008	0.996	0.98–1.01	-0.003	0.008	0.997	0.98–1.01
Sick leave days for elbow pain (periods >= 11 calendar days)[3)] (n=2 160 445 employee-months, n=75,962 employees)								
Immediate change in trend	-0.964	0.514	0.381	0.14–1.04	-0.398	0.486	0.672	0.26–1.74
Gradual change in trend	-0.034	0.072	0.967	0.84–1.11	-0.079	0.084	0.924	0.78–1.09
Linear trend before intervention (Slope)	0.003	0.014	1.003	0.98–1.03	-0.001	0.018	0.999	0.96–1.04

OHS=Occupational health care services, DC=diagnostic code. OR=Odds ratio, CI=Confidence interval. *p < 0.05, **p < 0.01, *** p< 0.001.

[1)] Immediate intervention estimate: Odds ratio was estimated for any employee to have any sick leave day after vs. before the intervention. Gradual interventionestimate: Odds ratio was estimated for any employee to have a sick leave day at index month vs. preceding month during the period after the intervention.[2)] Follow-up from 1st January 2013 to 21 st April 2017 [3)] Follow-up from 1st January 2013 to 31st December 2019. Adjusted for age, sex, occupational class, job contract, prescribed reimbursed purchases of medication for chronic diseases, prescribed reimbursed psychotropic medication.

For peer review only - http://bmjopen.bmj.com/site/about/guidelines.xhtml

References

1. *Tilastotietokanta Kelasto (Statistics in Finnish)*. [cited 2020 20.10.]; Available from: https://www.kela.fi/kelasto.
2. van Tulder, M., et al., *Chapter 3. European guidelines for the management of acute nonspecific low back pain in primary care.* Eur Spine J, 2006. **15 Suppl 2**: p. S169-91.
3. Costa-Black, K.M., et al., *Back pain and work.* Best Pract Res Clin Rheumatol, 2010. **24**(2): p. 227-40.
4. Black, C.M., *Sickness absence and musculoskeletal disorders.* Rheumatology (Oxford), 2012. **51**(2): p. 204-5.
5. Lotters, F.J., M. Foets, and A. Burdorf, *Work and health, a blind spot in curative healthcare? A pilot study.* J Occup Rehabil, 2011. **21**(3): p. 304-12.
6. Englund, L., G. Tibblin, and K. Svardsudd, *Variations in sick-listing practice among male and female physicians of different specialities based on case vignettes.* Scand J Prim Health Care, 2000. **18**(1): p. 48-52.
7. Arrelov, B.E., L. Borgquist, and K.F. Svardsudd, *Influence of local structural factors on physicians' sick-listing practice: a population-based study.* Eur J Public Health, 2005. **15**(5): p. 470-4.
8. Kankaanpaa, A.T., J.K. Franck, and R.J. Tuominen, *Variations in primary care physicians' sick leave prescribing practices.* Eur J Public Health, 2012. **22**(1): p. 92-6.
9. Hinkka, K., et al., *Physicians' experiences with sickness absence certification in Finland.* Scand J Public Health, 2019. **47**(8): p. 859-866.
10. Lahelma, E., et al., *Cohort profile: the Helsinki Health Study.* Int J Epidemiol, 2013. **42**(3): p. 722-30.
11. Sumanen, H., et al., *Age-group differences in sickness absence among the employees of the City of Helsinki 2002–2016 (Article in Finnish).* Finnish Medical Journal 2018. **73**(7): p. 438-44.
12. Gissler, M. and J. Haukka, *Finnish health and social welfare registers in epidemiological research.* Norsk Epidemiologi, 2004. **14**(1): p. 113-120
13. Harkko, J., et al., *Socioeconomic Differences in Occupational Health Service Utilization and Sickness Absence Due to Mental Disorders: A Register-Based Retrospective Cohort Study.* Int J Environ Res Public Health, 2020. **17**(6).
14. Martimo, K.-P. and J. Mäkitalo, *The status of occupational health services in Finland and the role of the Finnish Institute of Occupational Health in the development of occupational health services. Report for the international evaluation of the Finnish Institute of Occupational Health (FIOH)*. 2014, Finnish Institute of Occupational Health: Helsinki.
15. Lopez Bernal, J., S. Cummins, and A. Gasparrini, *Interrupted time series regression for the evaluation of public health interventions: a tutorial.* Int J Epidemiol, 2017. **46**(1): p. 348-355.
16. Lopez Bernal, J., S. Cummins, and A. Gasparrini, *The use of controls in interrupted time series studies of public health interventions.* Int J Epidemiol, 2018. **47**(6): p. 2082-2093.
17. Degli Esposti, M., et al., *Can synthetic controls improve causal inference in interrupted time series evaluations of public health interventions?* Int J Epidemiol, 2020.
18. Hojsgaard, S., U. Halekoh, and J. Yan, *The R Package Geepack for Generalized Estimating Equations.* Journal of Statistical Software, 2006. **15**(2): p. 1-11.
19. Gelman, A. and J. Hill, *Data Analysis Using Regression and Multilevel/Hierarchical Models*. 2007: Cambridge University Press.

For peer review only - http://bmjopen.bmj.com/site/about/guidelines.xhtml

20. Xiao, H., O. Augusto, and B.H. Wagenaar, *Reflection on modern methods: a common error in the segmented regression parameterization of interrupted time-series analyses.* Int J Epidemiol, 2020.
21. Shumway, R.H. and D.S. Stoffer, *Time Series Analysis and Its Applications: With R Examples*. 4th ed. 2017: Springer.
22. *Official statistics of Finland: Index of wage and salary earnings 2018, 3rd quarter*. 2018; Available from: https://www.stat.fi/til/ati/2018/03/index_en.html.
23. Rossignol, M., et al., *Coordination of primary health care for back pain. A randomized controlled trial.* Spine (Phila Pa 1976), 2000. **25**(2): p. 251-8; discussion 258-9.
24. Becker, A., et al., *Effects of two guideline implementation strategies on patient outcomes in primary care: a cluster randomized controlled trial.* Spine (Phila Pa 1976), 2008. **33**(5): p. 473-80.
25. Bussieres, A.E., et al., *Impact of imaging guidelines on X-ray use among American provider network chiropractors: interrupted time series analysis.* Spine J, 2014. **14**(8): p. 1501-9.
26. Leece, P., et al., *Improving opioid guideline adherence: evaluation of a multifaceted, theory-informed pilot intervention for family physicians.* BMJ Open, 2020. **10**(1): p. e032167.
27. French, S.D., et al., *Evaluation of a theory-informed implementation intervention for the management of acute low back pain in general medical practice: the IMPLEMENT cluster randomised trial.* PLoS One, 2013. **8**(6): p. e65471.
28. Al Zoubi, F.M., et al., *The effectiveness of interventions designed to increase the uptake of clinical practice guidelines and best practices among musculoskeletal professionals: a systematic review.* BMC Health Serv Res, 2018. **18**(1): p. 435.
29. Zhelev, Z., et al., *Effectiveness of interventions to reduce ordering of thyroid function tests: a systematic review.* BMJ Open, 2016. **6**(6): p. e010065.
30. Timmermans, S. and A. Mauck, *The promises and pitfalls of evidence-based medicine.* Health Aff (Millwood), 2005. **24**(1): p. 18-28.
31. McCluskey, S., A.K. Burton, and C.J. Main, *The implementation of occupational health guidelines principles for reducing sickness absence due to musculoskeletal disorders.* Occup Med (Lond), 2006. **56**(4): p. 237-42.
32. Buyle, F., et al., *Implementation of guidelines for sequential therapy with fluoroquinolones in a Belgian hospital.* Pharm World Sci, 2010. **32**(3): p. 404-10.
33. Shadish, W.R., T.D. Cook, and D.T. Campbell, *Quasi-experimental experiments: Interrupted time-series designs*, in *Experimental and quasi-experimental designs for generalized causal inference*. 2002, Houghton Mifflin Company: Boston, New York. p. 623.

For peer review only - http://bmjopen.bmj.com/site/about/guidelines.xhtml

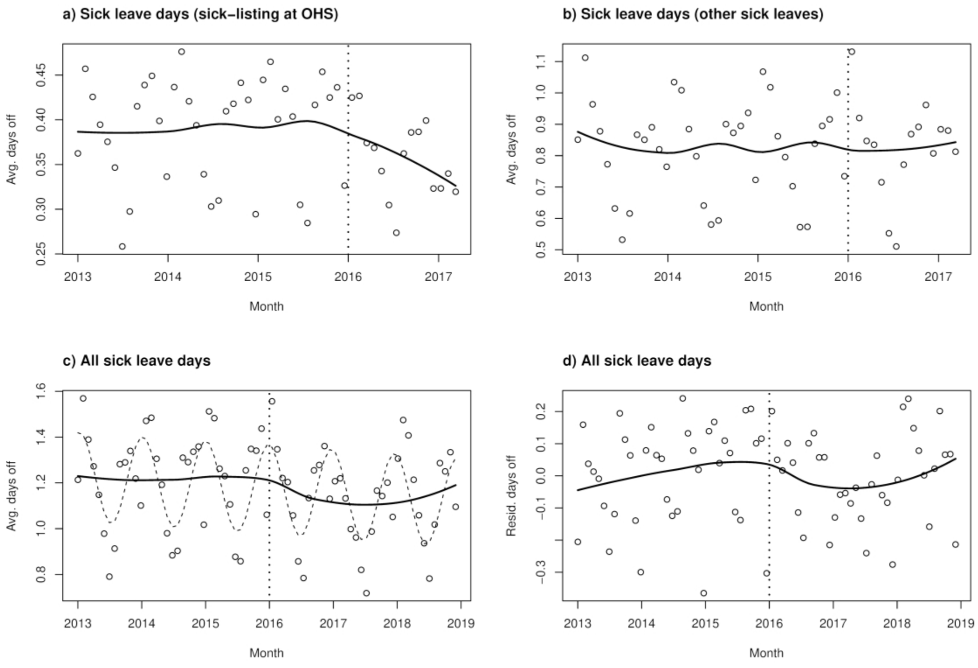

Figure 1: Model fits. Monthly averages of the number of all sick leave days. Intervention status could be specified until 21st April 2017 (Figures a) and b)). In Figures c) and d) the intervention status is not differentiated. Vertical dashed lines indicate the time point of intervention (all figures). Circles denote to observed averages, thick lines to their local regression fits (smoothed averages) (all figures), and thin dashed line to annual periodic variation (Figure c)).

173x120mm (800 x 800 DPI)

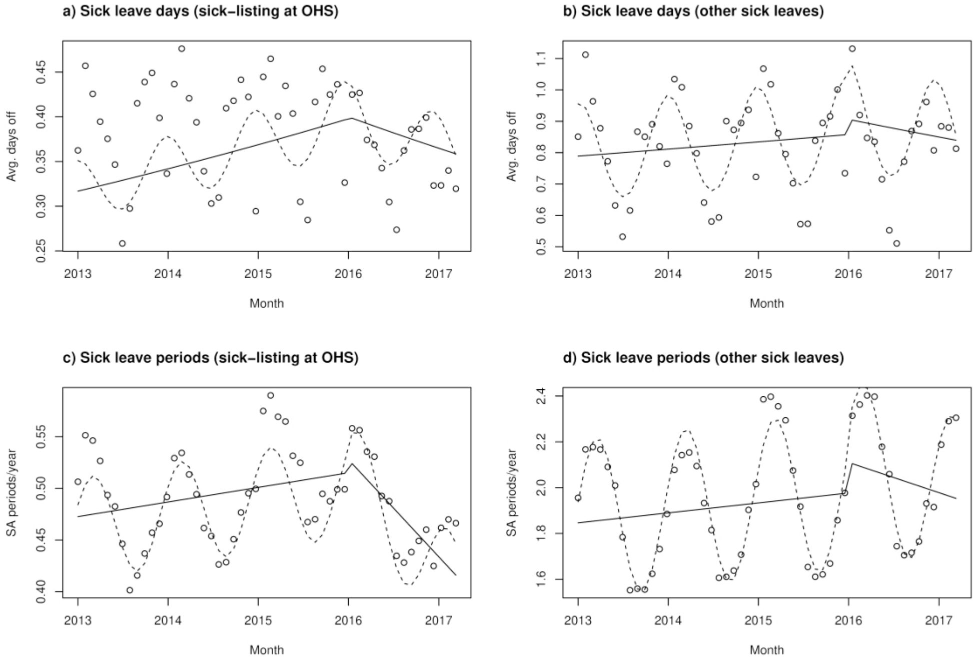

Figure 2: Model fits. Monthly averages of the number of all sick leave days (figures a) and b)) and periods (figures c) and d)). Observed data points denote to simple monthly averages and the lines represent GEE-model fits to employee-level data. The dashed line represents the full model prediction, including annual variations, whereas the solid line is the ITS-part of the model.
ITS=Interrupted time series analysis; GEE= generalised estimation equation

173x120mm (800 x 800 DPI)

Low back (lumbar back) pain, lumbago, M54.5

Lumbago is a very common ailment that everyone experiences at some point in their lives. It involves pain, aching and stiffness in the lumbar back. **It is almost always a harmless and benign condition**, which gets better by itself, but often recurs in following months. However, even if it recurs, its prognosis is good. The pain may begin suddenly in a harmless situation but may also develop gradually.

- **As a rule, lumbago gets better in a few days**. For more than 90% of sufferers, back pain is completely gone within 4–6 weeks.
- Signs of serious back disorders (continuous and progressively increasing backache even at rest, fever, weight loss, high-energy trauma, osteoporosis, changes in urinary and bowel continence, cancer history) can be ruled out using simple methods.

Assessing the need for sick leave:

○ According to new recommendations, sick leave may not at all necessary. There is no medical evidence that physically strenuous work slows down the healing of ordinary back pain or that it is associated with a risk of the condition continuing or of further injury.

○ **If sick leave is deemed necessary**: The patient's ability to do physically light work (only some lifting, bending and turning movements) may be **reduced for a few days or a week at the most.**

○ The patient's ability to perform physically strenuous work (which includes lifting, bending and turning movements) may be reduced for **two weeks at the most** (+ 2 weeks of partial sick leave if necessary), mainly because of difficulty lifting heavy objects. **Only after this does the checklist below apply.**

- **Temporary reduction and modification of pain-provoking factors are recommended.**
- Despite low back pain, working is not dangerous, and as a rule, continuing to work and not avoiding physical strain speeds up healing.
  - **Patients should be advised to use their backs normally – this is not dangerous**. Pain may become prolonged if the patient remains passive and afraid to move. Physical activity is beneficial; excessive resting is harmful. Moderate pain during exercise is not dangerous.
  - It is advisable to initially treat pain with sufficiently high doses of paracetamol and/or anti-inflammatory drugs. Strong painkillers (opiates) or muscle relaxants have not shown to be beneficial.
  - If the pain becomes prolonged, manipulation, massage or acupuncture may be beneficial.
  - In cases of prolonged pain, diverse drug-free pain management and pain threshold medicine can reduce pain sensitivity and improve pain tolerance.
  - Sleep and mood problems related to pain should be treated.

For peer review only - http://bmjopen.bmj.com/site/about/guidelines.xhtml

Checklist for when perceived work disability becomes prolonged:

DIAGNOSIS:

- Have **inflammatory diseases** (e.g., ankylosing spondylitis: Marie-Strümpell disease) been ruled out?
- Could the reason for the pain be a significant back **injury or previous cancer**?
- Does the pain or numbness radiate below the knee (**sciatic nerve entrapment)**?

WORK:

- Has work been modified to **temporarily reduce repeated lifting, leaning forward and twisted back postures, and work tasks that cause vibration**?
- Have p**sychosocial work load factors** (poor workplace atmosphere, lack of support from supervisor, excessive time pressure, overly demanding work, etc.) been identified? If so, has any attempt been made to change them?
- Has the employee **spoken to their supervisor** about their work and work ability?
- Have **occupational health services** been in contact with the supervisor **about work arrangements**?
- Has an **occupational physiotherapist** visited the workplace to advise on how to modify work tasks?

INDIVIDUAL FACTORS:

- Does the patient have any loading factors **outside of work** that may affect pain tolerance and coping with pain (crisis, grief, prolonged stress)?
- Does the patient do any **activities or** have any **hobbies outside of work** that may maintain the pain (sports, home renovations, long-distance driving, frequent use of laptop or mobile phone, gardening)?

TREATMENT:

- Have the **treatments** that the patient feels have best **benefit** been determined? Which treatments **make the pain worse**?
- Has the patient received **self-care instructions**?
- Does the patient use **active means** to control their pain? Exercise?

For peer review only - http://bmjopen.bmj.com/site/about/guidelines.xhtml

These issues, ‘yellow flags’, increase the risk of prolonged pain and work disability in the case of back pain:

- Sleep problems
- Anxiety, depression
- Fear of permanent damage or worsening of pain
- Pain all over the body
- Passivity, avoidance behaviour
- Problems at work, poor job satisfaction

For peer review only

For peer review only - http://bmjopen.bmj.com/site/about/guidelines.xhtml

Reporting checklist for cohort study.

Based on the STROBE cohort guidelines.

Instructions to authors

Complete this checklist by entering the page numbers from your manuscript where readers will find each of the items listed below.

Your article may not currently address all the items on the checklist. Please modify your text to include the missing information. If you are certain that an item does not apply, please write "n/a" and provide a short explanation.

Upload your completed checklist as an extra file when you submit to a journal.

In your methods section, say that you used the STROBE cohortreporting guidelines, and cite them as:

von Elm E, Altman DG, Egger M, Pocock SJ, Gotzsche PC, Vandenbroucke JP. The Strengthening the Reporting of Observational Studies in Epidemiology (STROBE) Statement: guidelines for reporting observational studies.

For peer review only

		Reporting Item	Page Number
**Title and abstract**			
Title	#1a	Indicate the study's design with a commonly used term in the title or the abstract	1

Abstract	#1b	Provide in the abstract an informative and balanced summary of what was done and what was found	2
**Introduction**			
Background / rationale	#2	Explain the scientific background and rationale for the investigation being reported	4
Objectives	#3	State specific objectives, including any prespecified hypotheses	5
**Methods**			
Study design	#4	Present key elements of study design early in the paper	5
Setting	#5	Describe the setting, locations, and relevant dates, including periods of recruitment, exposure, follow-up, and data collection	6
Eligibility criteria	#6a	Give the eligibility criteria, and the sources and methods of selection of participants. Describe methods of follow-up.	5
Eligibility criteria	#6b	For matched studies, give matching criteria and number of exposed and unexposed	n/a
Variables	#7	Clearly define all outcomes, exposures, predictors, potential confounders, and effect modifiers. Give diagnostic criteria, if applicable	9
Data sources / measurement	#8	For each variable of interest give sources of data and details of methods of assessment (measurement). Describe comparability of assessment methods if there is more than	5, 6, 9

For peer review only

		one group. Give information separately for for exposed and unexposed groups if applicable.	
Bias	#9	Describe any efforts to address potential sources of bias	10
Study size	#10	Explain how the study size was arrived at	5
Quantitative variables	#11	Explain how quantitative variables were handled in the analyses. If applicable, describe which groupings were chosen, and why	9
Statistical methods	#12a	Describe all statistical methods, including those used to control for confounding	10
Statistical methods	#12b	Describe any methods used to examine subgroups and interactions	10
Statistical methods	#12c	Explain how missing data were addressed	n/a
Statistical methods	#12d	If applicable, explain how loss to follow-up was addressed	n/a
Statistical methods	#12e	Describe any sensitivity analyses	12
**Results**			
Participants	#13a	Report numbers of individuals at each stage of study—eg numbers potentially eligible, examined for eligibility, confirmed eligible, included in the study, completing follow-	12

For peer review only

		up, and analysed. Give information separately for for exposed and unexposed groups if applicable.	
Participants	#13b	Give reasons for non-participation at each stage	n/a
Participants	#13c	Consider use of a flow diagram	n/a
Descriptive data	#14a	Give characteristics of study participants (eg demographic, clinical, social) and information on exposures and potential confounders. Give information separately for exposed and unexposed groups if applicable.	21
Descriptive data	#14b	Indicate number of participants with missing data for each variable of interest	n/a
Descriptive data	#14c	Summarise follow-up time (eg, average and total amount)	12
Outcome data	#15	Report numbers of outcome events or summary measures over time. Give information separately for exposed and unexposed groups if applicable.	22
Main results	#16a	Give unadjusted estimates and, if applicable, confounder-adjusted estimates and their precision (eg, 95% confidence interval). Make clear which confounders were adjusted for and why they were included	23
Main results	#16b	Report category boundaries when continuous variables were categorized	n/a
Main results	#16c	If relevant, consider translating estimates of relative risk into absolute risk for a meaningful time period	n/a

For peer review only

Other analyses	#17	Report other analyses done—e.g., analyses of subgroups and interactions, and sensitivity analyses	12, 23
**Discussion**			
Key results	#18	Summarise key results with reference to study objectives	16
Limitations	#19	Discuss limitations of the study, taking into account sources of potential bias or imprecision. Discuss both direction and magnitude of any potential bias.	18
Interpretation	#20	Give a cautious overall interpretation considering objectives, limitations, multiplicity of analyses, results from similar studies, and other relevant evidence.	17
Generalisability	#21	Discuss the generalisability (external validity) of the study results	17
**Other Information**			
Funding	#22	Give the source of funding and the role of the funders for the present study and, if applicable, for the original study on which the present article is based	16–19

The STROBE checklist is distributed under the terms of the Creative Commons Attribution License CC-BY. This checklist was completed on 12. November 2020 using https://www.goodreports.org/, a tool made by the EQUATOR Network in collaboration with Penelope.ai

For peer review only
